# Survey: Vulnerability Analysis of Low-Cost ECC-Based RFID Protocols against Wireless and Side-Channel Attacks

**DOI:** 10.3390/s21175824

**Published:** 2021-08-30

**Authors:** Souhir Gabsi, Vincent Beroulle, Yann Kieffer, Hiep Manh Dao, Yassin Kortli, Belgacem Hamdi

**Affiliations:** 1 Electronic and Micro-Electronic Laboratory, Faculty of Sciences of Monastir, University of Monastir, Monastir 5019, Tunisia; yassin.kortli@isen-ouest.yncrea.fr (Y.K.); belgacem.hamdi@gmail.com (B.H.); 2 LCIS Laboratory, Grenoble INP, University Grenoble Alpes, 26000 Valence, France; Vincent.Beroulle@lcis.grenoble-inp.fr (V.B.); yann.kieffer@esisar.grenoble-inp.fr (Y.K.); manh-hiep.dao@lcis.grenoble-inp.fr (H.M.D.)

**Keywords:** RFID, ECC, cryptography, lightweight, attacks, SCA

## Abstract

The radio frequency identification (RFID) system is one of the most important technologies of the Internet of Things (IoT) that tracks single or multiple objects. This technology is extensively used and attracts the attention of many researchers in various fields, including healthcare, supply chains, logistics, asset tracking, and so on. To reach the required security and confidentiality requirements for data transfer, elliptic curve cryptography (ECC) is a powerful solution, which ensures a tag/reader mutual authentication and guarantees data integrity. In this paper, we first review the most relevant ECC-based RFID authentication protocols, focusing on their security analysis and operational performances. We compare the various lightweight ECC primitive implementations designed for RFID applications in terms of occupied area and power consumption. Then, we highlight the security threats that can be encountered considering both network attacks and side-channel attacks and analyze the security effectiveness of RFID authentication protocols against such types of attacks. For this purpose, we classify the different threats that can target an ECC-based RFID system. After that, we present the most promising ECC-based protocols released during 2014–2021 by underlining their advantages and disadvantages. Finally, we perform a comparative study between the different protocols mentioned regarding network and side-channel attacks, as well as their implementation costs to find the optimal one to use in future works.

## 1. Introduction

RFID is an acronym for radio frequency identification. It indeed refers to a technology that can remotely identify objects or people. Besides, this technology utilizes electromagnetic fields to identify RFID tags that are naturally appended to objects. It is a very promising technology in terms of locating an object using real-time tracking. Its applications become wider when it comes to work along with IoT, where the combination of different devices works to collect the data from different sources. The technology for RFID tags is growing continuously. In the last years, RFID has been applied throughout industry and services, thanks to its low cost, ease of use, and its multiple practical applications, including healthcare, object identification, access control, passport verification, transportation and payment cards, car access control, supply chain traceability, logistics, or fee payments. However, despite becoming an everyday technology, many public and private entities have not considered the security of RFID systems as a basic requirement. In fact, like most electronics and networks, RFID systems are susceptible to many attacks and contain critical security flaws and vulnerabilities that allow for cloning tags or for straight signal replaying. Such vulnerabilities let attackers access certain services or facilities, get or alter personal information, and even track people. Thus, providing protection for these networks is essential, and security is one of the most critical issues facing these RFID systems [1]. 

On the other hand, wireless RFID tag attacks, among others, are particularly threatening.

The most known wireless attacks that hackers can perform on an RFID system are replay attacks, impersonation attacks, denial-of-service attacks, man-in-the-middle attacks, and tracking attacks [2]. The use of encryption and cryptographic primitives is necessary to avoid these attacks and guarantee privacy and data protection. There are mainly two encryption techniques: symmetric encryption and asymmetric encryption. Although there are key management issues with symmetric encryption (i.e., private-key cryptography), it is faster and functions without a lot of overheads on network or CPU resources and less power consumption. However, since symmetric cryptography uses the same secret key for data encryption and data decryption, this implies that along an RFID protocol all tags must share their secret key with all the readers. Without a secure channel for this secret key exchange, the tags are vulnerable to cloning attacks. To avoid this major problem, many authors proposed to use asymmetric cryptography that simplifies the problem of key management [3]. Among asymmetric cryptography techniques, ECC (elliptic curve cryptography) encryption techniques, based on the scalar multiplication operation, are comparatively faster and less complex asymmetric cryptography techniques. In recent years, several RFID authentication protocols using ECC were proposed. To respect the limited resources of RFID tags, the implementations of such primitives need to be low-power and low-cost. ECC cryptosystems implementations designed for low-resource and low-cost applications are called lightweight implementations [4]. To differentiate, a lightweight ECC implementation corresponds to an optimized implementation in terms of areas and hardware resources, while a lightweight RFID protocol [5] refers to protocol that uses only cyclic redundancy checks (CRC) and random number generators (RNG). This is also efficient security protocol, which is not the purpose of this paper to study. 

The advantage of ECC-based RFID authentication protocols is the prevention of any kind of secret key sharing between the tag and the server. Thanks to the ECC primitives, the secret keys are transmitted in an encrypted form. This encryption method is protected by the discrete logarithm problem (DLP) principle. In addition to RFID authentication protocols, the literature shows other types of wireless communication protocols, such as the key agreement protocols. These protocols rely on the principle of secret key sharing in such a way that this key will be known by one or more entities [6]. Among the most famous key agreement protocols are those based on chaotic maps and user-defined protocols [7,8].

Xing-Yuan et al. proved in their paper [9] that the key agreement protocol proposed by Tseng et al. [10] cannot guarantee the anonymity of the user and is not secure against MITM and Bergamo attacks. Therefore, the analysis performed by Xing-Yuan indicated that the use of a hash function based on a chaotic map is insufficient to guarantee the security of a key agreement protocol.

In 2012, Gong et al. [11] proposed a key agreement protocol based on chaotic maps. This protocol is assumed to be robust against different types of attacks and provides mutual authentication. However, Xing-Yuan et al. showed in their paper [12] the security limitations that presents Gong’s protocol. They mentioned that Gong’s work suffers from key management problems due to secret key sharing during communication and does not respect clock synchronization issues.

In the majority of these protocols, it is not easy to ensure mutual authentication between the entities communicating with each other and to respect the clock synchronization. In some cases, the chaotic Chebyshev card can be vulnerable to the Bergamo attack. Since these protocols are based on the sharing of secret keys, the increase in the number of entities communicating with each other can pose key management problems [13].

Moreover, Xing-Yuan has shown in his paper [7] that in order to transmit a single message sample during a secure communication scheme, we need to use N chaotic samples. In this way, the use of the chaos theory in messages during a wireless communication clearly decreases the message transmission rate between the sender and the receiver.

The first RFID authentication protocol based on elliptic curves has been proposed by Tuyls and Batina in 2006 in [14]. This protocol is based on the Schnorr identification protocol [15]. The serial multiplier used by Tuyls and Batina for the arithmetic multiplication operation only demands 2.6 K gates area. All computations made by this protocol need only around 10 K gates. However, several studies, such as [16], have shown that this protocol is vulnerable to tracking attacks and does not ensure mutual authentication nor forward secrecy. For this reason, Lee et al. proposed in [16] in 2008 an improvement of this protocol. Later, the two protocols published in 2008 [17,18] showed that the protocol of Lee et al. is also vulnerable to tracking and counterfeiting attacks and cannot ensure mutual authentication.

In 2007, Batina et al. implemented in [19] a second RFID identification protocol based on Okamoto schema [20]. The Okamoto schema can be considered more security effective than the Schnorr if we use the improvement techniques presented in [21,22]. However, in terms of implementation, the RAM required for an Okamoto identification protocol is, twice or more, higher than that used by a Schnorr protocol [23]. Lee et al. also studied the security of Batina’s protocol in [16] and showed that this protocol remains vulnerable to tracking attack.

Later, in 2014, Liao et al. proposed in [24] a secure RFID mutual authentication protocol based on ECC and integrating a public-key transfer. With this mutual authentication protocol, the server and the tag mutually authenticate each other. This schema needs five-point multiplication operations and 0.32 s computational time on 5 MHz tags. 

Batina et al. proposed in [25] a lightweight ECC architecture that requires only 12 k gates by using Montgomery’s algorithm for the scalar multiplication operation. They have reduced the number of intermediate registers used in the scalar multiplication operation. Batina et al. have shown that it is possible to implement ECC with less space to meet the surface and power requirements of RFID systems. Wenger et al. in [26] used for the tag implementation a low-resource processor that supports ECC operations for less than 9 K gates with an 80-bit security level. This solution uses an optimized 16-bit microcontroller suitable for low-power applications. Its power consumption is about 3.2 μW for this application. More recently, Wenger in [27] made a comparison between three different low power wireless sensor node architectures able to realize the ECC. The first architecture is an area and speed-optimized software solution, the second is a dedicated hardware module and the third is based on a hardware accelerator mixed with a CPU called “drop-in architecture”. The drop-in architecture requires less area than the dedicated hardware module with the same speed, while, compared to the optimized software solution, the power consumption of the drop-in architecture is reduced by a factor of 12. The suitability of asymmetric systems for RFID is still an open research problem due to the limitations of tag costs, gate count, and power budget. Two aspects must be taken into account: on the one hand, the ECC architecture, and on the other hand, the asymmetric mutual authentication protocol.

The use of an ECC crypto-system in RFID systems is intended to guarantee confidentiality and mutual authentication and to ensue secure communication against various attacks: cloning, eavesdropping, tracking attacks [28]. Moreover, other hardware attacks target directly the hardware vulnerabilities of the encryption blocks embedded into the tag: side-channel analysis (SCA) attacks and fault attacks (FA) [29]. The application of SCA attacks to contactless devices such as RFID is more complex than for contact devices. Since passive RFID tags are remotely powered by the electromagnetic field generated by the reader, extracting power measurements requires the insertion of a resistor between the analog front-end and the digital circuit that performs the encryption. It is impossible to apply this principle to RFID systems because they are usually integrated on a single piece of silicon. This is why originally few works focused on side channel attacks on RFID tags. In 2006, Oren was the first researcher who demonstrated in [30] the possibility of applying power analysis attacks on UHF tags. During this attack, Oren et al. considered that the attacker does not require any physical contact with the attacked device. This way, the attacker becomes completely passive during the data transmission, making the attack hardly detectable. Then in 2007, Hutter et al. published in [31] the first paper that examined the effectiveness of SCA attacks on RFID devices powered by a 13.56 MHz frequency that implemented an AES cryptosystem. To achieve this success, Hutter proposed two approaches to measure the electromagnetic consumption of an RFID device. These two methods consisted of either separating the RFID chip from the antenna by inserting another antenna into the reader’s detection field [32], or in filtering the total measured signal to eliminate the 13.56 MHz carrier of the reader. 

The difficulty of applying SCA attacks remains a major problem for RFID devices, but not impractical. Therefore, several RFID authentication protocols incorporating symmetric encryption cryptographic primitives are proposed to prevent information leakages leading to SCA attacks [33]. Nevertheless, among the ECC-based authentication protocols, no protocol focuses on the security of the encryption blocks against SCA attacks. For this reason, our paper will focus on classifying the different RFID authentication protocols based on ECC crypto-systems and study the security of these protocols as well as of cryptographic primitives against wireless attacks, and SCA attacks.

The remainder of this paper is organized as follows: Section 2 describes the principle and the different types of RFID tags. Section 3 presents the different types of attacks that target ECC-based RFID protocols and the vulnerability criteria necessary to implement them successfully. In Section 4, we briefly introduce elliptic curve-based cryptosystems. Section 5 deals with lightweight implementations of ECC dedicated to RFID applications based on optimized hardware architectures. A description of the most recent ECC-based RFID authentication protocols is the subject of Section 6. Section 7 is dedicated to a comparative study between the different described RFID protocols in terms of performance and security. Finally, a conclusion is made in Section 8.

## 2. RFID Technology

### 2.1. Working Principle

**Figure 1:**
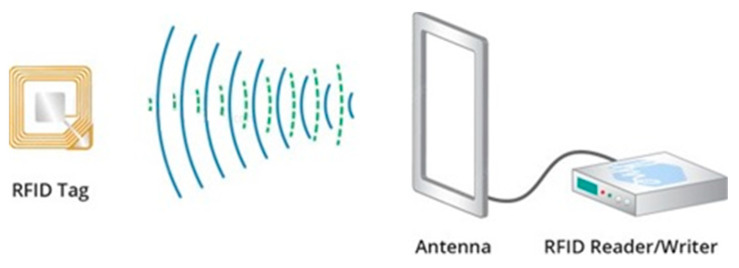
RFID system operation [34].

An RFID system ensures the communication between two entities: a reader and a tag. The reader allows identifying an object thanks to an RFID tag which is equipped with an electronic chip associated with an antenna [34]. The principle of operation of an RFID system is described in Figure 1. The reader sends a radio frequency signal to the tag it is trying to communicate with, and the tag responds in turn.

- The RFID reader is responsible for identifying the tag. It consists of a transmitter, a receiver, a microprocessor, and an antenna that sends an electromagnetic wave carrying a signal towards the element to be identified. In return, it receives the signals containing the information from the tags. The reader can be fixed or mobile, and its antenna can take several forms [35].
- The RFID tag, associated with the identified element, includes an electronic chip with a memory containing a unique EPC (electronic product code) identifier. Besides, to communicate to the reader in a given frequency band [35], the chip connects to an antenna.

### 2.2. RFID Tag Types

Depending on the power source and how the response is sent to the base station, RFID tags are grouped into three main categories: actives tags, semi-actives tags, and passives tags.

- *Actives tags:* the actives tags are used when reading ranges are greater than 10 m, and they can achieve ranges in the order of 50 to 100 m. The active term comes from the fact that the tags embed a battery to power both its logic electronics and its transmitter. Therefore, this implementation enables the tags to respond in different frequencies in the transmitting and receiving channel; consequently, it is possible for the active tags to communicate full duplex.
  The presence of a battery makes data writing possible, with a memory of up to 10 Kbits. They are given blank and can be several times written, deleted, modified, and read. The frequencies used by active tags are in the 433 MHz band, as well as in the 2.45 GHz and 5.8 GHz bands. Among the disadvantages of active tags, the very high cost reduces their use in different applications [35].
- *Semi-actives tags:* similar to active tags, semi-active tags also contain an energy source, but they do not use their battery to emit signals. They act as passive tags at the communication level. However, their battery allows them to record data during the transport of merchandise (temperature change, etc.).
  The cost of this type of tag is, therefore, lower than active tags. Generally, this type of tag has a simple design; however, they have several disadvantages:
  ✓ Reliability: it is impossible to know if their batteries are still operational.
  ✓ Cost: the connection of their batteries with their circuits increases the cost compared to a passive tag.
  ✓ Environmental impact: their battery contains highly polluting substances [36].
- *Passives tags:* passive tags, unlike active tags, work thanks to the energy provided by the reader. They integrate a dipole antenna that allows it to receive electromagnetic radiation from the reader. This radiation gives the passive tags enough power to authenticate themselves to the reader by transmitting their unique identification code. These passive tags are programmed with unmodifiable data for a capacity of 32 to 128 bits. They provide much lower unit costs than other technologies.
  In most cases, they are provided blank to the user, who will write the identification information and place them on the object that needs to be traced. This information can be read during the subsequent life of the tag but cannot be modified or completed. Passive tags are cheap and have an unlimited lifetime.

Each type of tag has its advantages and disadvantages according to the criteria on which the market depends. Passive tags offer the best choice thanks to the compromise they present between cost and reading distance as well as performance in terms of speed [35].

### 2.3. Operating Frequency Bands

RFID tags operate in different frequency bands. The choice of the operating frequency of a tag depends on several factors, such as the type of tag: active or passive, the distance between the tag and the reader, as well as propagation problems in the environment in which the tag and the reader communicate [37]. According to these factors, the operating frequencies of RFID tags can be classified into four bands: low frequencies (LF), high frequencies (HF), and ultra-high frequencies (UHF) [34]. Table 1 summarizes the different frequency bands of RFID tags. 

### 2.4. Communication Initiation

There are two types of communication between the tag and the server: the first type is tag talks first (TTF), where the tag takes the initiative to speak and starts the communication. The second type is reader talks first (RTF), where the reader firstly interrogates the tag and begins the communication [38]. 

The RTF transaction allows detecting a large number of tags in an acceptable interval of time. This transaction is usually used for passive tags because while the reader initials the communication, the former also feeds the tag enough energy for responding to the reader. 

For a TTF transaction, once the tag is in the reader’s RF field, it transmits its signal first to communicate. This transaction provides speedy and less complex identification of the tag compared to RTF protocols. With respect to the implementation of the RFID protocol, TTF transactions can be targeted by interception attacks because the tags transmit their data without the presence of the reader. The adversary can easily listen to this transmission without needing to send a signal to the tag to verify its presence.

### 2.5. Application Domains

**Figure 2:**
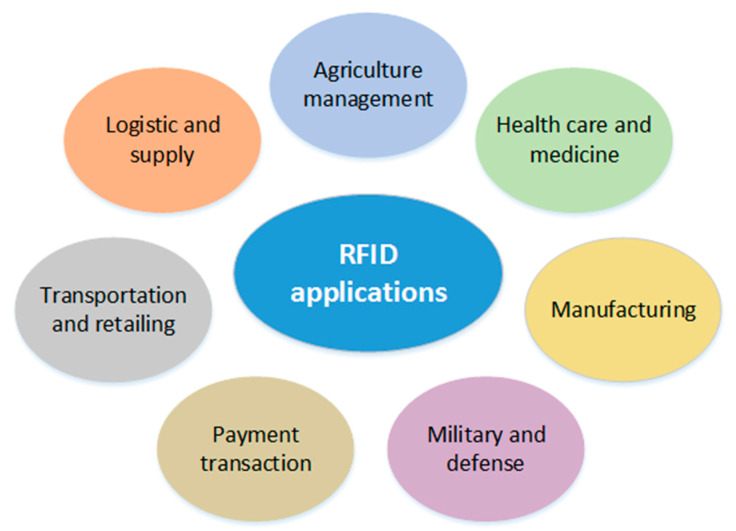
RFID application domains [38].

Passive RFID is a highly flexible system that can be used in a wide range of applications. Indeed, this technology facilitates the recognition and detection of different objects. Figure 2 below shows the various applications of RFID systems in our daily life. 

For example, in manufacturing, RFID systems that can resist extreme environmental conditions can be practical for controlling and monitoring operations and thus increase the efficiency of the manufacturing process [39,40,41]. RFID can be used to track the movement and health of animals [42]. In agriculture, it allows manual health tracking of all identified animals, automatically and without much expenditure [43,44]. More precisely, it helps to ensure that every animal on the farm is eating the right food. 

## 3. Security Attacks of RFID Protocols 

The security of the RFID authentication protocols relies on two main factors: the security of the RFID protocols and the security of the cryptographic primitives used to encrypt the processed data. Therefore, there are two main attack categories targeting the RFID authentication protocols: network attacks, also known as wireless attacks, which aim to attack the communication between the tag and the RFID reader, and the hardware attacks, which aim to break the encryption algorithm used in the RFID protocol. In this section, we will list and detail these two types of attacks.

### 3.1. Network Attacks

Like most electronic and network systems, RFID systems are vulnerable to several attacks that affect the reader to tag and the tag to reader communication. The goal of these attacks is to extract the secret identity of an RFID tag during RFID communication. An RFID protocol is said to be safe if it is secured and effective against different wireless attacks.

Possible attacks on an RFID system can be classified into three main groups: impersonation attacks, tracking attacks, and DoS (denial of service) attacks.

#### 3.1.1. Impersonation Attacks 

We talk about an impersonation attack when the attacker obtains either information related to the reader or information related to the tag to create an entity (reader/tag), then acts as a legitimate entity to proceed with the communication. Among the attacks that are classified as impersonation attacks, we can mention:

- *Eavesdropping attack:* the attacker is placed between the tag and the reader and listens to conversations to obtain important identification data. In this type of attack, the attacker is considered an unauthorized RFID reader [45].
- *Replay attack:* this attack is based on the principle of eavesdropping. After listening to the message, the attacker records a part of the conservation and replays it after a certain delay to the receiving device in order to steal information or gain access [46].
- *Relay attack:* the attacker is placed between the tag and the reader to relay word for word the message sent. The principle of this attack is that the two legitimate entities believe they are communicating directly with each other and do not realize that an illegitimate system is relaying between them.
- *Man in the middle attack (MITMA):* the attacker is placed between the tag and the reader to listen to the communication. Then he intercepts and manipulates the information. The attacker modifies the original signal and sends his incorrect signal while pretending to be a normal component in the RFID system.
- *Cloning attack:* this type of attack aims to imitate the identity of the tags. Indeed, the attacker borrows the identity of a reader, sends a request to the tag, then obtains the response from it. When the legitimate reader interrogates the tag, the attacker sends the response to the reader and identifies himself as the legitimate tag.
- *Server spoofing attack:* for this type of attack, the attacker presents himself as an authorized user of the system. The attacker impersonates a reader, sends a request to a tag, and then gets the response from the tag. When the legitimate reader queries the tag, the attacker sends the response to the reader to identify himself as the legitimate tag.

#### 3.1.2. Tracking Attacks

Tracking attacks are classified as system threats [47]. They are based on the weaknesses existing in the authentication protocol and the encryption algorithm. The attack consists of locating the tag and deducting its activity history. To do this, the attacker sends several requests to the tag, and by using the responses sent by the tag, he can easily determine where it is located. In fact, RFID tags are designed to always respond to different messages sent by the reader. If an attacker places himself in different locations and sends random messages to the tag, he receives the same response in different locations. The attacker can easily determine where the specific tag is currently located and which locations it has visited. At the same time, he cannot access the tag’s contents since he does not know its secret key. However, the adversary can use the fact that the tag always returns a constant response to the interrogations to make an illegal tracking and tracing.

#### 3.1.3. DoS Attacks 

DoS attacks are a category of attacks that can affect communication between legitimate tags and readers. The opponent sends several simultaneous signals to the server in the form of responses and makes the system unavailable for further communications. Among the DoS attacks, we can find:

- *Kill command attack:* it is a command used to disable the tag. The attacker issues more commands to permanently disable the tag [48].
- *Jamming:* since RFID tags listen to each radio signal within their range, an attacker can send electromagnetic signals in the form of noises to disrupt communication and prevent the tags from communicating with the reader [49].
- *Tag data modification:* DoS can cause the tag modification attack by allowing the attacker to modify the EPC (electronic product code) data on RFID tags to a random number that is not recognized by the reader [48].
- *De-synchronization attack:* this attack prevents the updating of secret quantities transmitted between the tag and the reader. A desynchronization attack is performed when the opponent can destroy the synchronous state between the tag and the server by blocking message updates which makes the values stored in the tag and the server different [49]. Indeed, a DoS attack could lead to a desynchronization attack.

### 3.2. Vulnerability Analysis to Network Attacks

As we mentioned earlier, the security of an RFID protocol is based on the security of the encryption primitive used. Network attacks aim to intercept the RFID communication between the tag and the reader in order to interact and get access to secret information. This type is called network attacks. The application of these attacks is possible if the legitimate server cannot control whether a tag is requested or not [50]. To ensure the confidentiality of secret data, RFID tags must not reveal information that can identify their bearer, such as their identifiers, their secret keys even during legitimate communications. So, among the criteria of the weakness of authentication protocols is the sharing of secret data that can give an attacker the ability to clone and relay the tags. In addition, in the context of access control, there is a risk of identity theft if the tags are not properly designed. The limited consumption and the restricted cost of the tags do not allow RFID authentication protocols to provide the same level of security [51]. This makes the comparison of the solutions much more difficult.

### 3.3. Security Requirements of RFID Systems

In addition to security against wireless attacks, RFID systems must provide certain security services [52] to ensure secure communication between the tag and the reader. Several previous research studies [53,54,55,56,57,58] have observed that to provide secure authentication, an RFID system should satisfy the following security requirements:

- *Mutual authentication:* during reader-tag communication, the attacker may react as a legitimate reader to obtain unauthorized information from the tag. Mutual authentication is the solution to this problem. Indeed, in addition to the authentication of the tag, the RFID system must also ensure the authentication of the reader. As a result, the reader and the tag authenticate each other.
- *Confidentiality:* to ensure data confidentiality, the identity of the tag must be secured and known only by the tag itself. Indeed, if an attacker obtains the tag’s identifier, he can easily trace its location and know its behavior. Confidentiality ensures that secret information cannot be obtained by an unauthorized user.
- *Anonymity:* the responses of tags should be randomized, so that it is infeasible to extract any information in communications between a tag and a reader.
- *Availability:* the variables communicated between the tag and the reader must be updated after each successful session. To ensure availability, the system must be successfully executed.
- *Forward security:* implies that the data transmitted from the tag must be independent and not linked to any other authentication session. This means that even if an attacker gets the current data from a tag, the past data remains secure and hidden, and the history of the tag’s movements remains known only by the tag.
- *Integrity:* this is translated into the fact that no private information is sent in clear text from the tag to guarantee the integrity of the messages transmitted between the reader and the tags. Data integrity is achieved by cryptographic systems based on elliptic curves.

### 3.4. Side-Channel Analysis Attacks

Side-channel analysis (SCA) attacks are the most powerful and famous hardware attacks against elliptic curves based crypto-processors. These attacks are based on information recovered during a hardware implementation of the cryptosystem execution on the circuit. This extracted information can be temporal information, electrical consumptions, and electromagnetic emanations. 

These attacks, also called hardware attacks, target the ECC cryptographic primitives used to encrypt transmitted data during a reader/tag communication in an RFID authentication protocol. During an ECC-based encryption system, the scalar multiplication of a point P by a scalar k, is a succession of addition and doubling operations that are chained together depending directly on the secret key used. SCA attacks use this dependency during the hardware implementation of the scalar multiplication operation to obtain the secret key k or a part of it.

In our paper, all discussed RFID protocols use elliptic curves as cryptographic primitives. For this reason, this section is dedicated to describing and presenting the most popular SCA attacks against elliptic curve cryptographic primitives.

#### 3.4.1. Timing Attack 

As mentioned in [59], timing attack uses differences in the execution times of certain cryptographic computations to deduce information about the secret key. Some cryptographic algorithms use conditional jumps that depend on the data being processed. The analysis of the execution times of these algorithms enables us to obtain secret information. The timing attack is assumed to be a passive attack, as it is based only on the observation of time needed to execute a certain calculation [59]. For example, the double-and-add scalar multiplication algorithm is susceptible to this type of attack. In fact, this algorithm performs a constant number of doubling operations on each execution, which is the number of bits of the private key used. However, the number of addition operations performed is equal to the number of bits “1” of the private key. It is therefore very simple to determine the number of non-zero bits (Hamming weight) of this key by an analysis of the computation time of the algorithm [60].

#### 3.4.2. Power Attacks

Power analysis attacks exploit potential correlations between the obtained power consumption traces and secret information manipulated during execution. These attacks are often divided into two categories: simple power analysis (SPA) attacks that require a single power consumption trace, and differential power analysis (DPA) attacks that use statistical tools between several power consumption traces.

- *Simple Power Analysis (SPA):* a SPA attack is based on the observation of the current consumption produced (or the electromagnetic radiation emitted) during a single execution of the targeted algorithm. This observation allows the attacker to deduce the information about the private by analyzing the consumption of extracted trace [61]. When calculating scalar multiplication, if the addition and doubling formulas are different, the attacker can easily differentiate them on a consumption trace. For example, when performing scalar multiplication with the double-and-add algorithm, we can find the private key bits used by distinguishing the power consumed by the doubling operation from the one consumed by the addition operation. The timing analysis attack against the double-and-add algorithm allows only to find the Hamming weight of the private key, while a SPA attack enables the retrieval of all the bits of the scalar. In addition, the calculation period of the doubling operation is half of the addition period. By analyzing a single trace of a scalar multiplication execution, the attacker can easily distinguish each operation used and determine the secret key’s value.
- *Differential Power Analysis (DPA):* the implementation of a DPA attack requires the collection of several consumption traces of a scalar multiplication operation using the same secret key. These types of SCA attacks use statistical analysis on a large number of samples to reduce noise by performing average calculations. For this reason, they are sometimes named statistical attacks [62]. The DPA attack requires knowledge of the computational algorithm used and a large amount of data to understand the relationship between the energy consumption of this processed data and the private key. During the scalar multiplication operation, the attacker needs a large number of power consumption traces $T_{i}$ for different points $P_{i}$ using the same private key k. By performing a statistical analysis of the processed data used and the corresponding consumption traces collected, the attacker can succeed to recover a part or the whole private key used [63]. The general principle of DPA is as follows [64]:
  - First, the attacker must choose a manageable part of the key, and then he conducts statistical analysis for any value that can take that manageable part.
  - For each encryption operation, the instantaneous consumption of the device is recorded.
  In fact, we can divide the implementation of this attack into two main phases: data acquisition and data exploitation.
  1. *Data acquisition*: during this phase, the processed data must be recorded many times. These data can be either cipher-texts or plain-texts. For example, if you want to extract N consumption traces, then, N cipher-texts or N plain-texts are registered. In addition, the N power consumption of the device during the encryption operations must be saved. Consequently, we could obtain a set of N pairs (M,T), where the pairs M and T are, respectively, the number of plain-text or cipher-text and the number of recorded traces of each operation.
  2. *Data exploitation:* the application of the attack requires the choice of a selection function and the sub-block of the attacked circuit. The result of this selection function must depend on known data and the secret key. Once this selection function has been chosen, it is necessary to divide the curves into two subsets $S_{0}$ and $S_{1}$. This distribution function is generally the Hamming weight of the output of the selection function or the value of one of its bits. For each possible value of the key at the input of the selection function, the set of traces S in input is separated according to the distribution function. For each of these distributions, the bias of the differential analysis is determined as the difference of the averages of the current curves over the two subsets. The key is determined by the assumption that generated the bias curve with the highest peaks. If none of the curves is different from the others, the attack has failed; this may be due to the insufficient number of traces [65].
- *Correlation Power Analysis (CPA):* the CPA attack is an improvement of the DPA attack previously explained. The statistical tool used in this attack is the Pearson correlation coefficient. This Pearson coefficient is used to determine the compatibility between two elements. The operating principle of this attack is based on the dependency between the current consumption of the circuit and the Hamming distance of the manipulated data. The CPA attack is based on the assumption that data leakage through an auxiliary channel depends on the number of bit variations from one state to another at a given time [66]. When applying the algorithm that produces the predictable result R, the attacker calculates the Hamming distance H between R and the various mi messages. Subsequently, the Pearson Correlation coefficient is calculated between the hamming distance matrix H and the consumption trace matrix T. According to this model, the Pearson coefficient for the calculation of the correlation ρ between T and H is given by the following formula:
  $$\rho_{\left(T,H\right)}=\frac{cov\left(T,H\right)}{\sigma_{T}\sigma_{H}}$$
  where H is the Hamming distance matrix of the model output for the 256 possible sub-keys $K_{j}$, cov is the covariance between T and H, and $(\sigma_{T}$, $\sigma_{H})$ are the standard deviations of T and H respectively [67]. Therefore, the correct key is the one that maximizes the correlations between current consumption and Hamming distance.

### 3.5. Vulnerability Analysis to Hardware Attacks

In this section, we will study the essential conditions that make the implementation of hardware attacks on the elliptic curve primitives, presented previously, successful. The knowledge of these factors can help us to avoid the realization of side-channel attacks on scalar multiplication algorithms and implement them safely in RFID protocols.

In observation attacks, the adversary can get information about the secret key by exploiting the circuit behavior, on the condition that the physical parameters processed depend on the secret data [66]. The use of conditional registers depending on the secret key and the knowledge of the addition and doubling operations formulas are among the main criteria of the vulnerability of elliptic curves to observation attacks. The implementation of SPA attacks requires a single execution of the calculation algorithm. The difference in consumption between the doubling and addition operations is the main factor of the success of this attack. Nevertheless, with DPA attacks, the attacker is required to repeat the calculation of the target algorithm several times using the same secret key. Even with the use of unified addition and doubling operations, the knowledge of the scalar multiplication algorithm used, the knowledge of the inputs/outputs of the algorithm, and the synchronization between the consumption traces of the different inputs are the critical factors for a successful DPA attack on elliptic curves [68].

The success factors of observation attacks (SPA/DPA/CPA) can therefore be summarized as follows [62]:

- Know either the inputs or the outputs.
- Execute a certain cryptographic algorithm that uses a certain unknown secret key.
- Use the same secret key for each execution.
- Know a cryptographic device model to estimate certain intermediate values that are related to the secret key.
- Estimate a part of the secret key.

## 4. Elliptic Curve Cryptography

### 4.1. Introduction

An elliptic curve, defined over the finite field $F_{q}\;$ [69], is a set of solutions (x,y) of a so-called Weierstrass equation:

(1) $$E:y^{2}+a_{1}xy+a_{3}y=x^{3}+a_{2}x^{2}+a_{4}x+a_{6}$$

where $a_{1}$, $a_{2}$, $a_{3}$, $a_{4}$, $a_{6}$ ∈ $F_{q}$. This equation can be simplified according to the characteristic (char) of the field ($F_{q}$) [69]:

- If char  ≥ 5, then $F_{q}=Fp,\;p$ is a large prime number, and the equation of the curve is given in [69] by:
  (2) $$E:y^{2}=x^{3}+ax+b\;with\;4a^{3}+27b^{2}\neq 0$$
- If char =3, then $F_{q}=Fp$, p is a prime number, and the equationof the curve, presented in [69], is given by:
  (3) $$E:y^{2}=x^{3}+ax+b\;with\;a^{3}b\neq 0\;$$
- If char =2, then = and the curve equation, given in [69], becomes:
  (4) $$E:y^{2}+xy=x^{3}+ax^{2}+b\;with\;b\neq 0\;$$

The most known finite fields for elliptic curves are prime fields having a characteristic strictly superior to three and binary fields with the characteristic equal to two [70].

### 4.2. Group Lows

Let $P\left(x_{P},y_{P}\right)$ and $Q\left(x_{Q},y_{Q}\right)$ are two points on the curve $E\left(F_{2^{m}}\right)$ and O the point at infinity, the group laws of this curve are as follows [70]:

- We have P+O=O+P=P for any point P ∈ $E\left(F_{2^{m}}\right)$.
- The opposite of point P is the point –P of coordinates $(x_{P},\;+{\;}_{}$, with P+(−P)=O.
- If P and Q are not opposed, then P+Q=R with:
  (5) $$x_{R}=\lambda^{2}+\lambda +a_{2}+x_{P}+x_{Q}$$
  (6) $$y_{R}=\left(\lambda +1\right).\;x_{R}+\lambda .\;x_{P}+y_{P}$$
  with:
  (7) $$\lambda =\left(y_{P}+y_{Q}\right)/\left(x_{P}+x_{Q}\right)\;\mathrm{if}\;x_{P}\;\neq \;x_{Q}$$
  (8) $$\lambda =x_{P}+\left(y_{P}/x_{P}\right)\;\mathrm{if}\;x_{P}=x_{Q}$$

### 4.3. Scalar Multiplication

In an elliptic curve, a multiplication between two points of the curve cannot be performed. Using a succession of addition and doubling operations, it is possible to define the multiplication of a point of the curve by an integer. This operation is known by scalar multiplication.

For any integer n∈N, the multiplication of the point P  by an integer n is defined by n.P=P+P+⋯+P, n times.

Scalar multiplication is the main operation of cryptosystems based on elliptic curves. The security of this operation relies on the fact that knowing P and n, we can easily compute Q=[n]P, but knowing P and Q it becomes difficult to find the integer n which verifies the equation [n]P=Q. This property is related to the discrete logarithm problem (DLP) [71].

An efficient implementation of scalar multiplication requires several decisions concerning: the selection of the finite field at the arithmetic level, the type of elliptic curve used to perform scalar multiplication, and the choice of the coordinate system used for the points representation. By ensuring the appropriate choice of these parameters, we can achieve a feasible implementation of elliptic curves adapted to the constrained devices [72].

### 4.4. Elliptic Curve Suitable for Low-Cost Applications

#### 4.4.1. Choice of Finite Field

In order to optimize the implementation of scalar multiplication, it is necessary to reduce the number of arithmetic operations used. These arithmetic operations depend on the field where the curve is defined. Therefore, it is necessary to choose the finite field which offers a suitable implementation for low-cost applications with easy and less expensive arithmetic operations in terms of hardware resources. The two well-known finite fields for elliptic curves are prime field and binary finite field.

However, elliptic curves can be defined on a prime field $\left(F_{q}\right)$, where q=p by the Equation (2). The elements of the prime field are integers between 0 and ([0,p−1]), where p is a prime number, and all field operations are computed modulo p. Indeed, the arithmetic operations on $F_{p}$, with p odd, need to propagate the carry throughout the calculation of addition, multiplication, or inversion. 

Elliptic curves defined on the binary fields $\left(F_{q}\right)$ where $q=2^{m}$ are presented by the curves of Equation (4). The elements of binary fields $F_{2^{m}}$ are polynomials of degree (m−1) with coefficients in $F_{2}:\left\{0,1\right\}$. So, each element of $F_{2^{m}}$ is represented as $A=\overset{m-1}{\underset{i=0}{\sum }}a_{i}.x^{i}$. Cryptosystems using the elliptic curves defined on $F_{2^{m}}$ must comply with certain requirements to ensure better security. However, such curves are used less and less because the $F_{2^{m}}$ field is considered too structured. Still, calculations on such cryptosystems have the advantage of being easier to implement.

Fournier recently indicated in his paper [73] that prime fields are preferred to binary fields because he claims that the discrete logarithm problem for binary elliptic curves can be solved using sub-exponential algorithms. Although, on the other hand, carry propagations by arithmetic operations in prime fields can be a source of weaknesses against side-channel attacks. In addition, despite the fact that the NIST Draft-800–186 standard [74] indicated that binary curves are depreciated due to their limited use by industry, Fournier showed that binary elliptic curves are more suitable than prime curves for implementation with low-cost devices. For these reasons, Fournier decided to choose binary elliptic curves for the implementation of IoT applications.

#### 4.4.2. Elliptic Curve Forms

The complexity of doubling and addition algorithms in terms of required arithmetic operations, depends on the choice of the finite field $F_{q}$. To accelerate the calculations of the scalar multiplication operations, it is necessary to use alternative models corresponding to each finite field. In this section, we present the different elliptic curve models defined on the binary and the prime fields and cite the advantages and disadvantages of each one.

- Elliptic curve forms over the prime field
  - *Montgomery curves:* the first type of elliptic curves defined on prime field ($F_{p}$) is the Montgomery model [75]. This model of curves is defined by the following equation [75]:
    (9) $$E_{A,B}:By^{2}=x^{3}+Ax^{2}+x$$
    where ($A,B)\in F_{p}$, B≠0, and $A^{2}\neq 4$. The advantage of using Montgomery curves is the possibility to implement them efficiently with the Montgomery-Ladder scalar multiplication algorithm. Therefore, the implementation of the Montgomery algorithm allows to speed up the calculation of the scalar multiplication through efficiency of the corresponding addition and doubling operations.
  - *Edwards curves:* The Edwards model presents an alternative form of elliptic curves, which admits a complete and uniform group law [76]. Either d or c are two elements of $F_{p}$, with d not squared, the Edwards curves are defined by the following equation [76]:
    (10) $$E_{d}:x^{2}+y^{2}=1+dx^{2}y^{2}$$
    The Edwards and Montgomery curves have the advantage of being bi-rational to a Weierstrass curve; this property is important in cryptographic applications, such as IoT [77]. For example, the calculation of the point exponentiation operation in an Edwards curve is 1.5 times more efficient than that performed in a Weierstrass curve [78].
  - *Twisted Edwards curves:* twisted Edwards curves are defined as a generalization of the Edwards curves. By incorporating a new parameter a, the equation of this curve looks as follows [76]:
    (11) $$E_{a,d}:ax^{2}+y^{2}=1+dx^{2}y^{2}$$
    where $\left(a,d\right)\in F_{p}$, d≠1, and a≠d. The twisted Edwards curves are the basis for the emergence of the EdDSA digital signature system, which offers high performance and prevents the security problems associated with other digital signature systems [79].
  - *Hessian curves:* Marc Joye et al. presented, in their paper [80], the Hessian curves defined by the following equation [80]:
    (12) $$E_{d}:x^{3}+y^{3}+1=dxy$$
    where $d\in F_{p}$, and d≠27. The advantage of using Hessian curves is that they are characterized by the use of unified formulas for addition and doubling of points in projective coordinates. This feature allows to avoid the possibility of applying SPA attacks.
  - *Huff curves:* Huff’s curves were proposed by Huff et al. in 1948 [81] and were later revisited by Joye et al. in 2010 [82] to have as final equation:
    (13) $$E_{a,b}:ax\left(y^{2}-1\right)=by\left(x^{2}-1\right)$$
    where $\left(a,b\right)\in F_{p}$, and $a^{2}\neq b^{2}$. Among the characteristics of Huff curves, the addition laws are complete and independent of the curve parameters. This addition law exhaustivity provides a natural protection against side-channel attacks [83].
- Elliptic curve forms over the binary field
  The majority of alternative models of elliptic curves in binary fields $F_{2^{m}}$ are an adaptation of pre-existing prime field models.
  - *Binary Edwards curves:* the Edwards binary curves present an adaptation of the Edwards curves defined on the prime field. They are proposed by Bernstein et al. [84] by the equation:
    (14) $$E_{d_{1},d_{2}}:d_{1}\left(x+y\right)+d_{2}\left(x^{2}+y^{2}\right)=xy+xy\left(x+y\right)+x^{2}y^{2}$$
    with $\left(d_{1},d_{2}\right)$ tow elements of $F_{2^{m}}$, such as $d_{1}\neq 0$ and $d_{2}\neq +$. An essential property of this curve model is its bi-rational equivalence with the Weierstrass model. This property allows to move from one model to the other and therefore ensures the compatibility of cryptographic protocols based on Edwards binary curves with those based on the Weierstrass curves.
  - *Binary Huff curves***:** in the same paper [82], Joye et al. also defined the equation of binary version of Huff’s curves by:
    (15) $$E_{a,b}:ax\left(y^{2}+y+1\right)=by\left(x^{2}+x+1\right)$$
    where $\left(a,b\right)\in F_{2}^{m}$ and a≠b. This curve form features a unified formula of addition and doubling point operations and a complete addition law, which makes this curve secure against certain side-channel attacks. Binary-Huff curves offer an efficient implementation due to the competitive arithmetic operations used. Devigne et al. showed in their paper [85] that every binary Huff form can be represented as a Weierstrass curve by the following equation:
    (16) $$E:v\left(v+\left(a+b\right)u\right)=u\left(u+a^{2}\right)\left(u+b^{2}\right)$$
    where $u=\left(\frac{ab}{xy}\right)\;\mathrm{and}\;v=\left(\frac{ab\left(axy+b\right)}{x^{2}y}\right).\;$ However, the opposite case is not always possible, in fact, not all binary curves can be expressed as a Huff curve.
    *Binary Hessian curves:* in binary fields $F_{2^{m}}$, the Hessian curves are defined by their generalized equation presented in [80] by:
    (17) $$E_{d,c}:x^{3}+y^{3}+c=dxy$$
    where c≠0 and $d^{3}\neq 27c$. Farashahi et al. showed in [80] that this form of curves supports complete and unified addition and doubling formulas, which means that the addition formulas are applicable to every input pair. Moreover, the point addition formulas of generalized binary Hessian curves are very fast and very efficient compared to those of the Hessian curves defined in prime fields.

#### 4.4.3. Point Representation System

The two fundamental operations of an elliptic curve are addition and doubling of points. These two operations depend mainly on the type of coordinate system used to present a point P on the curve E. The first coordinate system used in the literature is the affine representation. The analysis of the addition and doubling formulas using this system gives that each operation requires 1I+2M+2S, where I, M, and S present the inversion, multiplication, and square operations, respectively. The computational performance of this coordinate system is incompatible with the requirements of low-cost applications due to the high cost of the inversion operation. To avoid the high cost of this operation, the developers decided to replace the affine coordinate system with the projective coordinate system, which integrates a third coordinate Z. A projective coordinate system converts the coordinates (x,y) of a point P  by (X,Y,Z), where X=x, Y=y, and Z=1. Since affine coordinates require an inversion operation for each addition and doubling operation, projective coordinates have the advantage of using one single inversion operation to perform the entire scalar multiplication calculation. This single inversion operation is performed at the end of the scalar multiplication algorithm to re-convert the final result into affine coordinates. The conversion from projective coordinates to affine coordinates is performed by. x=X/Z and y=Y/Z. There are three main categories of projective coordinates: standard projective coordinates, Lopez and Dahab coordinates, and Jacobian coordinates. These three systems differ in the number of arithmetic operations used to perform addition and doubling operations. Bernstein et al. proposed in their paper [84] a unique point representation system called w-coordinates. This representation allows to replace the x and y-coordinates of point a point P by a single term w, such as w=x+y. Like the affine coordinate system, this representation requires several inversion operations for the calculation of addition and doubling operations. To avoid using this large number of inversion operations, the solution is to apply the conversion to the so-called projective-W coordinates system. The w-coordinate presentation has the advantage of reducing storage requirements and improving the efficiency of the main operations. But on the other hand, the conversion from the w-coordinate representation to the affine representation requires the use of a very expensive function called the half trace function [86]. Ideally, the most appropriate coordinate system is the one that will perform the minimum number of operations to calculate an addition and a doubling. Table 2 summarizes the use intensity of each coordinate system by the different research works published in the literature. From this table, we can find that the majority of the studied works have shown that the Lopez and Dahab coordinates implemented in binary fields are the least expensive in terms of the number of operations required for the scalar multiplication calculation [87]. For this reason, the Lopez and Dahab coordinates present the best choice to be adopted in order to achieve a scalar multiplication implementation suitable for constrained applications.

## 5. Lightweight ECC Implementations

Initially, RFID authentication protocols are based on symmetric encryption algorithms to keep the communication between the tag and the server properly secured. On the other hand, to prevent vulnerability to a specific type of attack, RFID tags need key exchange protocols. These services are generally provided by asymmetric cryptosystems. More recently, researchers started using public-key cryptosystems (PKC), provided that their hardware requirements are compatible with the limited resources of RFID applications. To achieve this goal, it is recommended to use elliptic curves-based cryptosystems. The ECC, with a key size of 160 bits, provides the same security level as an RSA cryptosystem with a key size of 1024 bits. This property makes the ECC the most attractive PKC for RFID devices. 

As discussed in the previous section, elliptic curves can be adapted to low-cost applications by setting the critical parameters at the arithmetic level [100]. At the hardware implementation level, the influencing factor in optimizing the implementation of scalar multiplication algorithms is the choice of the hardware architecture used. An adequate architecture allows obtaining results in conformity with the limited resources of RFID applications.

In this section, we will present the different ECC lightweight implementation architectures dedicated to RFID applications. The purpose is to determine the minimum number of gates needed to provide lightweight RFID authentication based on ECC. In 2009, Kulseng et al. showed in [101] that low-cost passive RFID tags could only support around 4500 gates to implement a secure communication protocol. 

Batina has shown in her paper [25] that the ECC processor can be developed to be suitable for lightweight and low-power applications such as RFID. This paper presents a proposal for a low-power ECC processor over $F_{2^{131}}$. It needs only 6718 gates for the modular arithmetic logic unit and the control unit. This processor uses Montgomery’s algorithm for the implementation of scalar multiplication, which allows saving registers because the Montgomery algorithm uses only the x-coordinate in the affine representation. In fact, Batina showed the efficiency of its processor and the reduced number of necessary gates required compared to Kumar’s work [102], which requires a 12 K gate area complexity. Nevertheless, these results obtained by Batina do not take into account the data memory of the used processor.

Later, in 2008, Lee proposed in [103] one of the most efficient ECC-based solutions in terms of the area dedicated for low-cost applications. It consists of an elliptic curve processor (ECP) defined on $F_{2^{163}}$ using a small 8-bit microcontroller to support higher-level protocol implementations. This processor requires 12.5 Kgates and around 276 K cycles to execute a single scalar multiplication operation. These synthesis results do not take into account the ROM and RAM consumption needed for data storage, which can influence the total processor implementation area required.

Using the same key size as Lee et al. 163 bits, Wenger has implemented in 2011 [26] a new ECC processor on $F_{2^{163}}$ that requires arround 8958 gate of total area and 285 K cycles to perform one scalar multiplication operation. This processor is a combination of a 16-bit multi-precision architecture and an area-optimized 16-bit custom microcontroller. The 16-bit microcontroller provides flexibility to be adapted to various applications. Wenger demonstrated in his article that this combination significantly minimizes the required area of macro RAM blocks and avoids processor clutter by handling data by 16-bit blocks. All these advantages make this implementation an improvement of about 4% gate area compared to the supposed best solution of Bock et al. [104].

In 2013, Wenger published a new paper [27] that focuses on a comparative study between three ECC-based architectures. The first architecture consists of a software solution optimized in terms of area and speed. The second architecture corresponds to a hardware module, and the third one is a new “drop-in” ECC architecture. All three architectures use an open MSP430 system [105], which is an important factor in saving data memory. The advantage of using the Open MSP430 model is to avoid loading constants before they are used in the RAM memory, which is supposed to be very expensive. The results obtained by Wenger show that the module of the first software solution requires between 16 K and 14 K gates, while the second optimized hardware module uses a minimum of 11,778 gates without considering the area that requires the MSP430. With regard to the third solution, the ECC drop-in module presents the most efficient solution in terms of the number of gates needed since it only uses between 4114 and 6760 gates. Therefore, the ECC drop-in architecture presents an interesting solution for low-cost applications.

Roy is also interested in the lightweight implementation of elliptic curves for low-cost applications. He proposed in [106] a lightweight coprocessor for a 16-bit microcontroller using 283-bit Koblitz curves. This proposal offers a 140-bit security level, and its implementation requires only a 4323 gate area. Azarderakhsh has shown in [107] that it is possible to accelerate the calculation speed of the scalar multiplication with Koblitz curves by representing the scalar as r-adic expansions. For this reason, Roy proposed a first lightweight scalar conversion algorithm implemented for the first time with Koblitz curves. However, the first use of Koblits curves to provide a lightweight implementation dedicated to low-cost applications was by Azarderakhsh in [107]. Azarderakhsh used the Koblitz curves defined in $F_{2^{163}}$ which requires 11,571 gate area. As a final result, the architecture of Roy et al. presents a decrease of about 64% in the area needed compared to that described in [107], with a higher level of security.

All the mentioned works justify the feasibility of developing an ECC lightweight implementation that is adaptable to the limited resource constraints of low-cost applications. It should be noted that most of these works use the elliptic curves defined on the binary fields $F_{2^{m}}$. It shows the efficiency of this field and its impact in reducing the number of gates required in ECC implementations. 

Table 3 summarizes the results obtained from the implementation of each work previously described in terms of area, number of cycles, and power/energy consumption. The total area required for the implementation of these works ranges from 4323 to 15,356 gates. 

Based on the results listed in Table 3, we can conclude that it is possible to have a lightweight ECC implementation compatible with limited resource applications if we can change the parameters that impact the total cost of major ECC operations. For example, as shown in Roy’s work, the choice of Koblitz curves with scalar conversion reduces the total processor area by 7248 gates compared to [107].

## 6. Analysis of Proposed ECC-Based RFID Protocols

We will start this section by presenting a detailed explanation of different RFID authentication protocols that have been published in the last years. All the proposed protocols are based on elliptic curve crypto-systems. Moreover, we are going to deal with the security failures that present each protocol to the different wireless and physical attacks.

### 6.1. Liao et al. Protocol

In 2014, Liao et al. proposed in [24] a secure RFID authentication system based on ECC integrated with ID-verifier transfer protocol. They indicated that their system is robust against various types of attacks, completely solves existing research problems, and meets the essential needs of an RFID system. Liao et al. have shown that this protocol is an improvement of Liu’s protocol [108], presented in 2013, by ensuring confidentiality and security against attacks: spoofing, cloning, and tracking. This protocol consists of two phases: the setup phase and the authentication phase. 

#### 6.1.1. Setup Phase 

In the setup phase, the server and the tag are equipped with the public parameters of the elliptic curves (q,a,b,P). The server chooses a random number $x_{S}$ as its private key and calculates its public key =P. Then, it chooses the quantity $x_{T}$ as the private key of the tag and calculates =P as the identifier or public key of the tag.

#### 6.1.2. Authentication Phase

**Figure 3:**
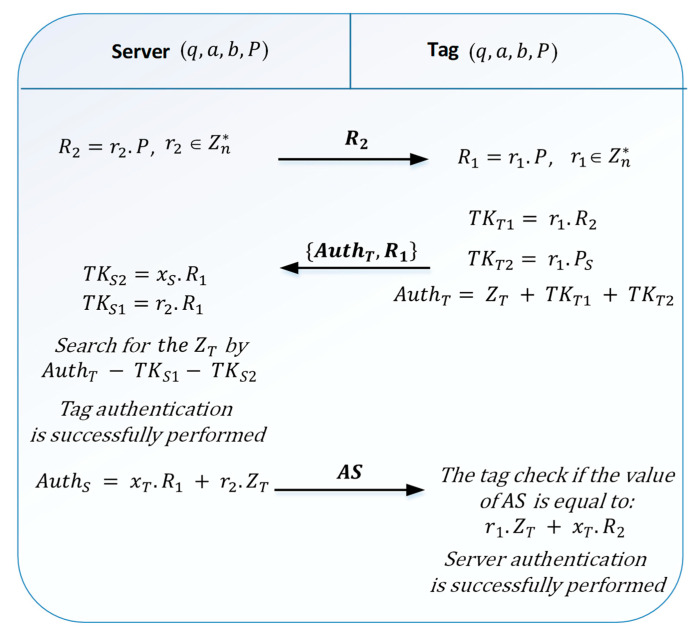
Liao’s authentication protocol.

The authentication phase of the Liao protocol is described in Figure 3. During this phase, the server and the tag communicate with each other according to the following steps:

- *Step1*: the server randomly chooses a number $r_{2}$ and calculates =P. Then, it sends the value of $R_{2}$ to the tag.
- *Step2:* when $R_{2}$ is received, the tag, in turn, chooses a random number $r_{1}$ and calculates =P. The tag also calculates two temporary secret keys $=R_{2}$ and $=P_{S}$. To encrypt the value of $Z_{T}$, the tag then calculates the quantity $={+}_{}+$ and sends $Auth_{T}$ and $R_{1}$ to the server.
- *Step3:* the server calculates its temporary keys $=R_{1}$ and $=R_{1}$. It uses these two keys to extract the value of $Z_{T}$ by the following equation:
  $$\begin{array}{rr} Auth_{T}-TK_{S1}- & TK_{S2}=Z_{T}+TK_{T1}+TK_{T2}-TK_{S1}-TK_{S2}\\ & =Z_{T}+r_{1}R_{2}+r_{1}Ps-r_{1}R_{1}-x_{S}R_{1}\\ & =Z_{T}+r_{1}r_{1}P+r_{1}x_{S}P-r_{1}r_{1}P-x_{S}r_{1}P=Z_{T} \end{array}$$
  Then, the reader searches for the value of the tag identifier in its database. If found, the reader confirms the validity of the tag and obtains the corresponding private key $x_{T}$. Then, the server calculates $Auth_{S}=x_{T}R_{1}+r_{2}Z_{T}$ and transmits $\left(Auth_{S}\right)$ to be authenticated by the tag.
- *Step4:* finally, the tag calculates the quantity $r_{1}+R_{2}$ and checks if the value is equal to the received $Auth_{S}$ value. If the two quantities are equal, the tag confirms that the server is authentic. As we can see, the Liao et al. protocol ensures mutual authentication between the server and the tag.

### 6.2. Zhao et al. Protocol

In his paper, Zhao et al. showed in [109] that Liao’s protocol is vulnerable to key compromise attacks. For this reason, they proposed a new ECC-based RFID protocol that meets the protocol security issues in [24]. The proposed protocol also consists of two phases: the setup phase and the authentication phase.

#### 6.2.1. Setup Phase 

The server and the tag generate their public and private keys during this phase. First, the server chooses a number $x_{S}$ as its private key and calculates its public key =P. Second, the server sets for each tag the secret key $x_{T}$ and calculates the corresponding public key =P. Finally, the server keeps $\left(x_{S},P_{S},x_{T},Z_{T}\right)$ in its database, and the keys $x_{T}$ and $Z_{T}$ in the tag memory.

#### 6.2.2. Authentication Phase 

The mutual authentication between the tag and the server is done according to the following steps:

- *Step1:* the server chooses a random number $r_{2}$, calculates $R_{2}=r_{2}P$, and sends the message $\left\{R_{2}\right\}$ to the tag.
- *Step2:* after receiving $R_{2}$, the tag also chooses a random number $r_{1}$ and calculates $R_{1}=r_{1}P=\left(k_{x},k_{y}\right)$. Then, it calculates its two temporary keys $Tk_{T1}=\left(r_{1}k_{x}\right)R_{2}$ and $Tk_{T2}=\left(r_{1}k_{y}\right)P_{S}$ and $Auth_{T}=Z_{T}+Tk_{T1}+Tk_{T2}$. The tag then sends the message $\left\{Auth_{T},R_{1}\right\}$ to the server.
- *Step3:* after receiving $Auth_{T}$ and $R_{1}$, the server calculates the two keys $Tk_{S1}=\left(r_{2}k_{x}\right)R1$ and $Tk_{S2}=\left(x_{S}k_{y}\right)R_{1}$ and checks if $Z_{T}=Auth_{T}-Tk_{S1}-Tk_{S2}$. Then, the server checks if the calculated $Z_{T}$ is in its database. If it was the case, the server obtains the value of $x_{T}$, calculates the quantity $Auth_{S}=x_{T}R_{1}+r_{2}Z_{T}$ and send the message $\left\{Auth_{S}\right\}$ to the tag. Else, the server stops the process.
- *Step4:* when receiving the message, the tag checks if the value of $Auth_{S}=r_{1}Z_{T}+x_{T}R_{2}$. If they are equal, the server is authentic; otherwise, the protocol stops.

### 6.3. Alamr et al. Protocol

In 2018, Alamr et al. proposed in [110] a new RFID authentication protocol based on elliptical curves that use the ECDH (elliptic curve Diffie-Hellman) protocol as a key exchange technique to establish secure communication between the tag and the reader. The ECDH protocol permits each party to have its own public-private key pair and to generate a new modifiable key that can be used to encrypt the communication. This protocol is based on the ECDLP and the elliptic curve factorization problem (ECFP). The ECFP is to find the [s]P and [t]P points of the quantity Q=[s]P+[t]P.

The principle of this protocol is divided into two phases: the setup phase and the authentication phase.

#### 6.3.1. Setup Phase 

First, the server selects a random number $Pr_{R}$ as the reader private key and =P as the reader public key. Second, the server chooses a random number $Pr_{T}$ as the tag’s private key and calculates =P as the tag’s public key. Then, the tag and the reader, each one keeps its private-public key pair and system parameters (P: base point, n: EC order).

#### 6.3.2. Authentication Phase 

**Figure 4:**
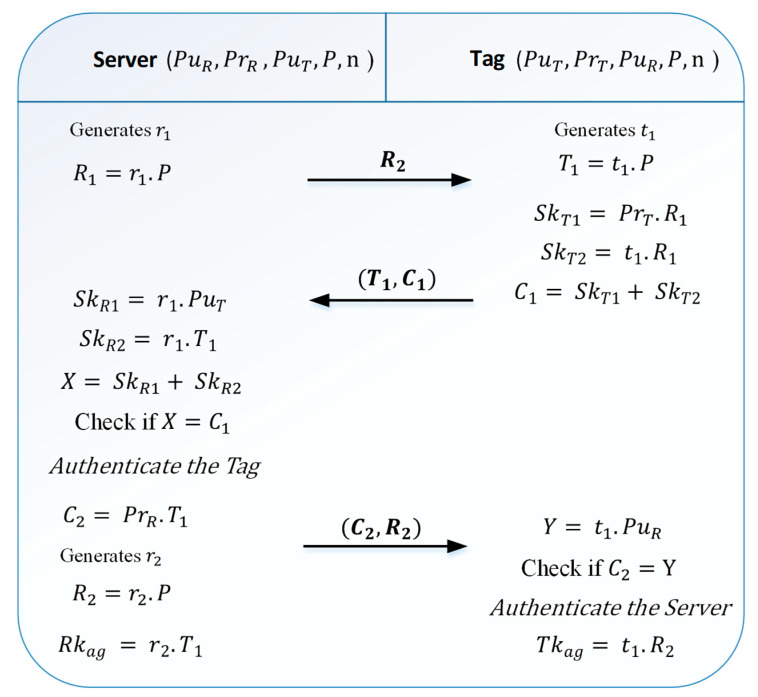
Alamr’s authentication protocol.

The authentication protocol process presented in Figure 4 is as follows:

- *Step1:* the reader generates a random number $r_{1}$ and calculates $R_{1}=r_{1}P$. Then, it sends the value of $R_{1}$ to the tag.
- *Step2:* after receiving $R_{1},$ the tag chooses a random number $t_{1}$ and calculates $T_{1}=t_{1}P$. Then the tag calculates its two secret keys; $Sk_{T1}=Pr_{T}R_{1}$ and $Sk_{T2}=t_{1}R_{1}$. Lastly, to encrypt its two secret keys, the tag calculates $C_{1}=Sk_{T1}+Sk_{T2}$ and sends the message $\left\{T_{1},C_{1}\right\}$ to the reader.
- *Step3*: the reader, after receiving $T_{1}$ and $C_{1}$, calculates its two temporary keys; $Sk_{R1}=r_{1}Pu_{T}$ and $Sk_{R2}=r_{1}T_{1}$. Then, it calculates $X=Sk_{R1}+Sk_{R2}$ and compares it with the value of $C_{1}$. If they are equal, the reader authenticates the tag, and then it calculates $C_{2}=Pr_{R}T_{1}$. After that, the reader generates a number $r_{2}$ and calculates $R_{2}=r_{2}P$ and it sends $C_{2}$ and $R_{2}$ to the tag.
- *Step4:* during this step, the tag calculates $Y=t_{1}Pu_{R}$ and compares it with the value of $C_{2}$. If they are equal, the tag authenticates the reader.
- *Step5:* at the end of this phase, the two entities fix the key agreement transmitted between them. The key agreement of the tag $Tk_{ag}=t_{1}R_{2}$ and this of the reader key $Rk_{ag}=r_{2}T_{1}$.

### 6.4. Naeem et al. Protocol

More recently, in 2019, Naeem et al. proposed in their paper [111] an enhancement to the ECC-based protocol of Alamr et al. This enhancement is considered safe and robust and can be deployed in any IoT environment. Performance analysis of this protocol shows that it is less costly in terms of resources required and more secure than the Alamr’s protocol. The operating process of this protocol consists of two phases: the setup phase and the authentication phase.

#### 6.4.1. Setup Phase 

The server generates all the system parameters. It first selects the identity of the tag. Then, it chooses the value $Pr_{R}$ as the secret key of the reader and calculates the point =P as its public key. At the end of this phase, the server stores in the reader database the values $\left\{X_{T},Pr_{R},Pu_{R}\right\}$ and in the tag database the values $\left\{X_{T},Pu_{R}\right\}$.

#### 6.4.2. Authentication Phase

Naeem’s protocol authentication process is detailed by the following steps:

- *Step1:* the reader generates a random number $r_{1}$ to calculate the point $R_{1}=r_{1}P$. Then, it sends the value of $R_{1}$ to the tag.
- *Step2:* the tag in its turn produces a random number $t_{1}$ and calculates $T_{1}=t_{1}P$. Then it calculates $C_{1}=t_{1}R_{1}$ and $C_{2}=X_{T}+h\left(T_{1},R_{1},C_{1}\right)$. Then the tag sends the message $\left\{C_{1},C_{2}\right\}$ to the reader.
- *Step3:* using the two quantities $C_{1}$ and $C_{2}$, the reader calculates $T_{1}={\left(r_{1}\right)}^{-1}C_{1}$ and $X_{T}=C_{2}-h\left(T_{1},R_{1},C_{1}\right)$ and it checks the value of $X_{T}$ in its database. If the value of $X_{T}$ calculated is equal to the one stored, the reader authenticates the tag and then calculates $C_{3}=Pr_{R}T_{1}$ and $C_{4}=h\left(C_{3},X_{T},T_{1},R_{1}\right)$. At the end of this step, the reader sends $C_{4}$ to the tag and calculates its key agreement $RK_{ag}=X_{T}r_{1}T_{1}$.
- *Step4:* when it receives $C_{4}$, the tag calculates $Y=t_{1}Pu_{R}$. If the value of $C_{4}$ is equal to $h\left(Y,X_{T},T_{1},R_{1}\right)$, the tag authenticates the reader. Consequently, if the authentication is successful, the tag calculates its key agreement $Tk_{ag}=X_{T}t_{1}R_{1}$.

### 6.5. Dinarvand et al. Protocol

In 2019, Dinarvand et al. proposed in [112] a mutual RFID authentication protocol based on elliptic curves, which aims to prevent and overcome the weaknesses of the various protocols previously proposed. Dinarvand has demonstrated that the proposed protocol meets the requirements of an RFID authentication protocol in terms of the number of resources, communication cost, and storage capacity. This protocol consists of three main phases: setup phase, authentication phase, and updating phase.

#### 6.5.1. Setup Phase 

During this phase, the server sets the public parameters of the curve and produces the public and private keys of the tag and its own. It chooses a random number $x_{S}$ as its private key and calculates =P as its public key. The server chose $x_{T}$, a point on the curve as the unique tag identifier. Then, the server selects an $ID_{S}$ number, randomly, as a pseudonym of the tag and sets a number K as the secret key shared between the tag and the server. At the end of this phase, the server stores $\left\{ID_{S},x_{T},K\right\}$ into its database, and $\left\{ID_{S},x_{T},P_{S},K\right\}$ in the tag memory.

#### 6.5.2. Authentication Phase 

During the authentication phase, the tag and server authenticate each other. Dinarvand et al. described the steps of this phase as follows:

- *Step1:* the server selects a random number $r_{1}$, calculates $R_{1}=r_{1}P$, and sends $R_{1}$ to the tag.
- *Step2:* the tag chooses a number $r_{2}$ to calculate $R_{2}=r_{2}P$ and sends the message $\left\{ID_{S},R_{2}\right\}$ to the server.
- *Step3*: as soon as it receives $ID_{S},$ the server searches this value in its database. If it finds it, the server takes the corresponding secret key K and the point $x_{T}$ from its database and calculates: $TK_{S1}=r_{1}KR_{2}$**,** $TK_{S2}=x_{S}KR_{2}$, and $Auth_{S}=TK_{S1}TK_{S2}x_{T}$, and sends the message $\left\{Auth_{S}\right\}$ to the tag. Otherwise, if the value of $ID_{S}$ is not in the database of the server, the corresponding tag is assumed invalid.
- *Step4:* after receiving $Auth_{S}$, the tag calculates $Tk_{T1}=r_{2}KR_{1}$, $TK_{T2}=r_{2}KP_{S}$, then it checks the equation:
  $${x_{T}}^{\prime }=Tk_{T1}⊕TK_{T2}⊕Auth_{S}$$
  If they are equal, the tag authenticates the server. Then, it calculates $Auth_{T}={x_{T}}^{\prime }2Tk_{T1}2TK_{T2}$ and sends it to the server.
- *Step5:* during this phase, the server checks if the received value $Auth_{T}$ is equal to $x_{T}2TK_{S1}2TK_{S2}$. If they are equal, the server authenticates the tag. Otherwise, the process stops.

#### 6.5.3. Updating Phase 

If the mutual authentication is successfully performed, the tag and server update their secret key K and the pseudonym of the tag $ID_{S}$. In this phase, the server should keep the old and the new $ID_{S}$ of each step.

- For the tag, the update of K and $ID_{S}$ is done as follows:
  $$ID_{S}{}^{*}=X\left(Tk_{T1}\right)⊕ID_{S}⊕K$$
  $$K^{*}=X\left(TK_{T2}\right)⊕2K$$
  $$ID_{S}=ID_{S}{}^{*}$$
  $$K=K^{*}$$
- And for the server, the update of K and $ID_{S}$ is as follows:
  If $ID_{S}{}^{old}$ is received:
  $$ID_{S}{}^{new}=X\left(TK_{S2}\right)ID_{S}{}^{old}K$$
  $$K^{new}=X\left(TK_{S2}\right)2K^{old}$$
- If $ID_{S}{}^{new}$ is received:
  $$\;ID_{S}{}^{old}=ID_{S}{}^{new}$$
  $$K^{old}=K^{new}$$
  $$ID_{S}{}^{new}=X\left(TK_{S2}\right)ID_{S}{}^{old}K$$
  $$K^{new}=X\left(TK_{S2}\right)2K^{old}$$

### 6.6. Benssalah et al. Protocol

Benssalah proposed in his paper [113] published in 2020 a new RFID authentication protocol based on ECC. This protocol presents a modification of Dinarvand’s protocol at the process level of the authentication phase. In fact, the protocol of Benssalah consists of two phases: the authentication phase and the updating phase. Initially, the server database and the tag database are stored successively by $\left\{x_{T},x_{S},ID_{S}\right\}$ and $\left\{x_{T},ID_{S},P_{S},P\right\}$.

#### 6.6.1. Authentication Phase

The authentication phase carried out between the tag and the server is divided into four steps, which are described as follows:

- *Step1:* the server chooses a random number $r_{1}$ and sends it directly to the tag.
- *Step2*: the tag chooses a random number $r_{2}$, then it calculates $R_{2}=r_{2}P_{S}$, $R_{3}=r_{2}P$, and $R_{4}=x_{T}+h\left({\left\{R_{2}\right\}}_{x}\left|\left|{\left\{R_{3}\right\}}_{x}\right|\right|r_{1}\right)$ and it sends, afterwards, $R_{3}$, $R_{4}$, and the $ID_{S}$ to the server.
- *Step3:* once it receives $R_{3}$, $R_{4}$, and $ID_{S}$, the server uses its secret key $x_{S}$ to compute $R_{2}^{*}=x_{S}R_{3}$ and $x_{T}=R_{4}-h({\left\{R_{2}^{*}\right\}}_{x}\left|\left|{\left\{R_{3}\right\}}_{x}\right|\right|\;r_{1})$. Then, based on the pseudonym $ID_{S}$ sent by the tag, the server looks for the value $x_{T}$ in its database to authenticate the tag. After that, the server computes $R_{5}=h(x_{T}\left|\left|{\left\{R_{2}\right\}}_{x}\right|\right|r_{1}||R_{4})$ and transmits it to the tag.
- *Step4:* the tag calculates $R_{5}^{*}=h(x_{T}\left|\left|{\left\{R_{2}\right\}}_{x}\right|\right|r_{1}||R_{4})$, then it compares it to the received $R_{5}$ value. If the two values are equal, the tag authenticates the server and updates the value of $ID_{S}$, otherwise, the authentication process is stopped.

#### 6.6.2. Updating Phase

When the tag and the server successfully authenticate each other, they update the tag’s $ID_{S}$ value to move to a new authentication session. The $ID_{S}$ updating steps at the tag and server level are given as follows:

- For the tag:
  $$ID_{S}{}^{*}=h({\left\{R_{2}\right\}}_{x}\left|\left|ID_{S}\right|\right|r_{1}||R_{4})$$
  $$ID_{S}=ID_{S}{}^{*}$$
- For the server:
  If $ID_{S}{}^{old}$ is received:
  $$ID_{S}{}^{new}=h({\left\{R_{2}\right\}}_{x}|\left|ID_{S}{}^{old}\right|\left|r_{1}\right||R_{4})$$
- If $ID_{S}{}^{new}$ is received:
  $$ID_{S}{}^{old}=ID_{S}{}^{new}$$
  $$ID_{S}{}^{new}=h\left({\left\{R_{2}\right\}}_{x}\left|\left|ID_{S}{}^{old}\right|\left|r_{1}\right|\right|R_{4}\right)$$

### 6.7. Zheng et al. Protocol 

Zheng et al. proposed in 2017 [114] an authentication protocol using elliptic curves. This protocol is proposed to be more secure against camouflage attacks and tracking attacks, and that ensures confidentiality, anonymity, and forward security. Considering that only the channel between the tag and reader is not safe, this protocol consists of two phases: the initialization phase and the authentication phase.

#### 6.7.1. Setup Phase 

During this phase, the server chooses a random number $S_{S}$ as its private key and calculates =P as its public key. The tag also chooses a random number $S_{T}$ as its private key and calculates =P. $P_{T}$ is assumed as the tag identity information.

The server keeps its private and public keys and the identity of the tag in its data base. At the same time, the tag keeps its private key, its identity information, and the public key of the server in its memory.

#### 6.7.2. Authentication Phase

- *Step1:* the server randomly chooses a number $r_{1}$ and calculates $R_{1}=r_{1}P$. It sends $R_{1}$ to the tag.
- *Step2:* the tag selects a random number $r_{2}$ and calculates $R_{2}=r_{2}P$, $AT=P_{T}+r_{2}P_{S}$, and $AT^{\prime }=S_{T}R_{1}-r_{2}R_{1}$, then it sends the message $\left\{R_{2},AT,AT^{\prime }\right\}$ to the server.
- *Step3:* the server calculates $P_{T}=AT–S_{S}R_{2}$ and searches for the tag based on the value of $P_{T}$ stored in its database. The server then checks if $AT^{\prime }=r_{1}P_{T}-r_{1}R_{2}$. If they are equal, the tag authentication is successfully performed; otherwise, the process stops.
- *Step4:* the server generates the value $AS=S_{S}R_{2}-r_{1}R_{2}$ and sends it to the tag.
- *Step5:* the tag checks if $AS=r_{2}P_{S}-r_{2}R_{1}$. If they are equal, the server authentication is performed; otherwise, the authentication does not pass.

### 6.8. Yang et al. Protocol

In its paper published in 2018 [115], Yang et al. proposed an improvement of Kaur’s RFID authentication protocol. The modified protocol aims to eliminate all security deficiencies in the Kaur protocol in order to provide more secure authentication. Yang et al. described a lightweight and improved anonymous authentication protocol for RFID systems using the elliptic curve cryptography algorithm. This protocol consists of two phases: the initialization phase and the authentication phase.

#### 6.8.1. Setup Phase 

During this phase, the server and tag save their public and private keys and the public system parameters. First, the server chooses a number $x_{S}$ as its private key and calculates =P as its public key. Second, the tag selects a number $x_{T}$ as its private key and calculates =P as its public key. Finally, the server must store its public and private keys and the identity ID of each tag into its database, and each tag saves its identity and public and private keys.

#### 6.8.2. Authentication Phase 

The authentication process is carried out in the following steps:

- *Step1:* the server, first, obtains a current temporary variable $ts_{1}$ and the identity ID of the tag that it wants to interrogate. Then, it calculates $Pid_{1}=H\left(ID,ts_{1}\right)$, $Auth_{S}=x_{S}X_{T}$ and $Ver_{S}=H(ts_{1},\;Pid_{1},\;Auth_{S}$). The server then transmits the message $M_{1}=\left\{ts_{1},Pid_{1},Ver_{S}\right\}$ to the tag.
- *Step2:* when it receives $M_{1}$, the tag first checks the freshness of the time variable $ts_{1}$. If $ts_{1}$ is over the set expiration time, the tag does not consider this message. Otherwise, the tag test if its identity checks the equation $Pid_{1}=H\left(ID,ts_{1}\right)$. If the ID identity checks the value of $Pid_{1}$, the authentication process continues.
- *Step3:* the tag calculates $Aut{h_{S}}^{\prime }=x_{T}X_{S}$ and tests if this value verifies the equation $Ver_{S}=H\left(ts_{1},Pid_{1},Aut{h_{S}}^{\prime }\right)$. If it’s, the server is authenticated by the tag. Otherwise, the tag treats the message $M_{1}$ as a modified message and deletes it.
- *Step4:* the tag then obtains the current time variable $ts_{2}$ and calculates $Pid_{2}=H\left(X_{T},ID,ts_{2}\right)$, $Auth_{T}=Aut{h_{S}}^{\prime }$, and $Ver_{T}=H(ts_{2},\;Pid_{2},\;Auth_{T}$). Then, it sends the message $M_{2}=\left\{ts_{2},Pid_{2},Ver_{T}\right\}$ to the server.
- *Step5:* as soon as the server receives the message $M_{2}$, it first checks the freshness of $ts_{2}$. Then it determines if the public key $X_{T}$ corresponding to the identity ID checks the equation $Pid_{2}=H\left(X_{T},\;ID,\;ts_{2}\right)$. If this assumption is verified, the equation $Ver_{T}=H\left(ts_{2},Pid_{2},Auth_{S}\right)$ is maintained. So, the tag is successfully authenticated by the server.

### 6.9. Alaoui et al. Protocol

Alaoui et al. proposed in [116] in 2021 two ECC-based RFID protocols that offer mutual authentication and resistance to the most significant security attacks. The first protocol requires storing a list of authorized tags and keys on the reader’s side, while the second protocol only requires storing the list of unauthorized tags on the reader. As a result, the two protocols differ in the storage requirements on the server side, but they perform the same security level against the different attacks. For this reason, we choose to describe in this section the protocol that requires storage on the reader’s side. The process calculation of this protocol is divided in two phases: initialization phase and authentication phase.

#### 6.9.1. Setup Phase 

This phase permits the tag to store its own identity $id_{n},$ two corresponding private keys $\left(K_{n1},K_{n2}\right)$ and the reader public key $Q_{r}=d_{r}.G$. On the other side, the reader stores the secret quantities associated to the tag ($id_{n}$, $K_{n1},K_{n2}$) and its pair of public and private keys respectively $Q_{r}$ and $d_{r}$.

#### 6.9.2. Authentication Phase 

The authentication process of this protocol is carried out according to the following steps:

- *Step1:* the server chooses a random number $r_{r}$ and computes $R_{r}=r_{r}.G$ = ($x_{r},\;y_{r})$ The server then transmits $R_{r}$ to the tag.
- *Step2:* the tag also chooses a random number $r_{n}$ to compute the point $R_{n}=(r_{n}+K_{n2}$).G. Then, in order to compute $A_{1}=K_{n1}⊕h_{r1}\;$, the tag derives the quantity $H\left(\left(r_{n},K_{n2}\right)\left(Q_{r},R_{r}\right)\right)=\left(h_{r1},h_{r2}\right)$. Next, the tag computes $H(id_{n}\left|\left|K_{n1}\right|\right|K_{n2}\left|\left|R_{r}\right|\right|R_{n}||h_{r2})=(h_{1}||h_{2})$ and transmits the message {$R_{n}$, $\;A_{1}$, $h_{2}$} to the reader.
- *Step3:* using its private key $d_{r}$ and the random generated number $r_{r}$, the reader calculates the quantity $\left(d_{r}+r_{r}\right).R_{n}=\left(h_{r1},h_{r2}\right)$ to find the tag secret key $K_{n1}=A_{1}⊕h_{r1}$ and searches it in its data base. If the reader cannot find any correspondence to this key in its database, the protocol stops. Otherwise, it recovers the identity $id_{n}$ of the tag related to this key and the second secret key $K_{n2}$ and computes $H(id_{n}\left|\left|K_{n1}\right|\right|K_{n2}\left|\left|R_{r}\right|\right|R_{n}||h_{r2})=(h_{1}||h_{2})$. If the calculated $h_{2}$ value matches the received $h_{2}$ value, the reader authorizes the request and sends $h_{1}$ to the tag.
- *Step4:* in the last step, the tag compares the stored $h_{1}$ value with the value received from the reader. If the two values are similar, the authentication is successfully approved, otherwise the tag quits the process.

### 6.10. Izza et al. Protocol 

In 2021, Izza et al. were concerned with the security of wireless communication systems through the proposition of their RFID authentication protocol [117] that meets the security limitation of Naeem [111] protocol. Izza et al. assert that their improved scheme achieves both scalability, security, and privacy for RFID systems. Izza assumed that, during this protocol, the communication channel between the reader and the server is insecure. This protocol consists of three major phases: initialization and registration phase, authentication phase, and digital signature and data transmission phase.

#### 6.10.1. Initialization Phase

This phase allows registering the secret data corresponding to the users, the tags, the readers and the medical server (MS). The tag pseudo identity, the server pseudo identity, the reader’s public key and the reader’s private key, the server’s public key, and the server’s private key, respectively $PID_{T}$, $PID_{R}$, $Pu_{R}$, $Pr_{R}$, $Pu_{S}$, and $Pr_{S}$ are stored in the database of network manager (NM).

#### 6.10.2. Authentication Phase

- *Step1:* the server generates a random number $r_{1}$ to calculate $R_{r1}=r_{r}.P$ and sends it to the tag $T_{i}$.
- *Step2:* when the tag receives $R_{r1}$, it first chooses a random number $t_{1}$ and calculates $C_{1}=t_{1}.P$ and $R_{t1}=t_{1}.Pu_{R}$. Then, the tag initializes the value $PID_{T_{i\;new}}=h(PID_{T_{i\;old}}||init)$ and calculate $C_{2}=PID_{T_{i\;new}}+h({\left(R_{t1}\right)}_{x}\left|\left|{\left(R_{r1}\right)}_{x}\right|\right|{\left(C_{1}\right)}_{x}||T_{1})$, where $T_{1}$ represents the current timestamp. At the end of this step, the tag transmits the messages {$C_{1}$, $\;C_{2}$, $\;T_{1}$} to the reader.
- *Step3:* after receiving the messages, the reader first checks the time spent. If the spent time is less than ΔT, the reader does not stop the session. Subsequently, using its private key, the reader extracts the tag’s pseudo identifier $PID_{T_{i\;new}}$ and search for it in its database. If the reader finds the identity of the tag in its database, the tag is successfully authenticated. Next, it calculates $R_{t1}^{*}=C_{1}.Pr_{R}$.
  Then, the reader communicates with the medical server (MS). it calculates the message $N_{1}=r_{1}.Pu_{S}$ and initializes $PID_{R_{\;new}}=h(PID_{R_{\;old}}||init)$ and $N_{2}=PID_{R_{\;new}}+h\left({\left(R_{r1}\right)}_{x}\left|\left|ID_{R}\right|\right|{\left(N_{1}\right)}_{x}T_{2}\right)$, where init is a random number selected by the MS and also inserted in the reader and tag memories during the initialization phase. The message {$N_{2}$, $\;R_{r1}$, $\;T_{2}$} is sent to the MS, where $T_{2}$ corresponds to the new timestamp.
- *Step4:* after authenticating the reader, the MS generates a random number $s_{1}$ and calculate $S_{1}=s_{1}.P$ and $R_{s1}=s_{1}.Pu_{R}$. Then, it replies to the reader with the messages: $T_{3}$ (the MS’s new timestamp), $S_{1}$, and $N_{3}=$ $h({\left(R_{s1}\right)}_{x}\left|\left|PID_{R}^{*}\right|\right|T_{2}||T_{3})+ID_{S}$. The reader receives the messages, checks the time interval, and authenticates the MS.
- *Step5*: by using the previous initialization of the pseudo identifier $PID_{R_{\;new}}=h(PID_{R_{\;old}}||init)$, the reader computes the message $C_{3}=h\left(ID_{Ti}\left|\left|T_{3}\right|\right|T_{4}\right)$ and the message $C_{4}=h({\left(R_{t1}^{*}\right)}_{x}\left|\left|PID_{R_{\;new}}\right|\right|{\left(R_{r1}\right)}_{x}||T_{4}).$ Next, the reader sends the quantities {$C_{3}$, $\;C_{4}$, $\;T_{3}$, $\;T_{4}$} to the tag and updates its pseudo-identifiers and those of the tag. Finally, the reader generates its own shared session key $SK_{RT}=h\left(ID_{Ti}\left|\left|PID_{T_{\;new}}\right|\right|{\left(r_{1}.C_{1}\right)}_{x}\right)$
- *Step6:* The tag further verifies the time interval $T_{5}-T_{4}$ and authenticates the reader. Finally, the tag generates an ephemeral session key $SK_{TR}=h\left(ID_{Ti}\left|\left|PID_{T_{\;new}}\right|\right|{\left(t_{1}.R_{r1}\right)}_{x}\right)$.

#### 6.10.3. Data Transmission Phase

- *Step1:* the tag generates a message $m_{i}$ and encrypts it using the shared key $SK_{TR}$. Then, the tag sends the message {$M_{i}=E_{SK}\left(m_{i}\right)$, $T_{5}$}.
- *Step2:* the reader finds mi with using its own $SK_{RT}$ session key. Subsequently, using the elliptic curve digital signature with message recovery (ECDSMR) mechanism, the reader shares the same message with the MS.

## 7. Comparative Study of ECC-Based Authentication Protocols: Implementation Cost and Vulnerability

All these protocols are based on elliptic curves, but they differ in their security criteria and implementation costs. As we saw in the previous section, all these protocols differed in the number of operations used by the tag and the server at each execution (scalar multiplication operations, number of point addition operations, number of hash functions, etc.) and in their effectiveness against the different wireless attacks. 

### 7.1. Implementation Cost

First, we will classify these protocols according to the number of operations used by the tag and by the reader at each execution. Table 4 shows the dependence of each protocol on the number of operations for the execution of a single authentication session. As also shown (Table 4), all these protocols differ in the number of operations used. Some protocols require random numbers, others require point addition operations, and some others use hash functions.

Even though all these protocols are based on elliptic curves, they do not all use the same number of operations. For this reason, we see that Dinarvand’s protocol uses three scalar multiplication operations for tag and reader, while the Liao, Zhao, and Naeem’s protocols require five scalar multiplications for the tag and five for the reader. Moreover, we can notice that the Yang protocol requires only one scalar multiplication operation for the tag and one scalar multiplication for the reader, but it uses four hash operations for the tag and four hash operations for the reader. Indeed, four hash operations in a single execution are very expensive in terms of resources and memories for an RFID tag. Izza indicates in his article that his protocol performs two scalar multiplications and six hash operations at the tag level, which requires a large storage area and a very important computation time. On the other hand, during the Aloui’s protocol execution, the tag uses two scalar multiplication operations and only two hash operations, which allows the reduction of the consumption cost compared to the Yang protocol.

In addition, Benssalah requires a total of six hash functions and four scalar multiplications in one round. Baashira indicated in his paper [118] that the use of cryptographic hash functions increases the level of protocol security but at the same time requires more computing capacity, which must be taken into account for applications with constrained resources. The number of scalar multiplication operations has an impact on the computational cost of an RFID communication. In fact, it is quite obvious that the computational time needed to perform a scalar multiplication operation is longer than the one needed to perform an addition operation since a scalar multiplication operation, using a scalar of size n bits, requires almost between n and n/2 addition operations during a single execution and n doubling operations.

Since RFID tags are limited hardware resources, an RFID system looks for solutions that do not require hash functions [52]. Moreover, Table 5 and Table 6 classify the proposed protocols in terms of computation cost and communication cost, respectively.

Considering that for Aloui’s protocol, the time required to execute a scalar multiplication operation, Tm, is equal to 37.94 ms. For all other protocols, the time needed to calculate a scalar multiplication operation, Tm, is 64 ms. The calculation time, presented in Table 5, is proportional to the number of scalar multiplication operations used in the authentication protocol multiplied by the time needed for a simple scalar multiplication execution. The Yang protocol uses the lowest number of scalar multiplication operations for the tag and for the reader. However, we can not claim that this protocol has the lowest computational cost since the execution of this protocol involves four hash operations at the tag level. In contrast, Dinarvand’s protocol uses only three scalar multiplication operations at the tag level, and two simple point addition operations, which can be considered as the lowest calculation cost among these different protocols. On the other hand, Aloui’s protocol is the most consuming one in terms of calculation cost, and it requires a total of 765.20 ms to execute all the protocol operations. In addition, Liao, Zhao, and Naeem’s protocols need a total of 640 ms to calculate the scalar multiplication operations required during the authentication.

For each of the considered protocols, the communication costs are obtained by calculating the length of all messages transmitted during the communication processes of an authentication protocol. As we can see from Table 6, the ECC-based protocols, Zhao, Zheng, and Dinarvand present a total communication cost equal to 1280 bits. This implies that the length of data transmitted through these three protocols is the smallest compared to the other protocols. However, for Izza’s protocol, all the transmitted data have a size of 256 bits, which results in a very high communication cost. We can therefore deduce that the Izaa’s protocol is the most consuming one in terms of communication costs.

From Table 5 and Table 6, it can be seen that Zhao, Zheng, and Dinarvand’s protocols present a good compromise between the computation time and the cost of communication compared to the rest of the protocols.

### 7.2. Security Analysis

In this subsection, we are interested in the security analysis of the different proposed protocols. Table 7 and Table 8 examine the security and vulnerability of different protocols to wireless attacks and physical attacks that can suffer an RFID system.

#### 7.2.1. Security against Wireless Attacks 

Table 7 shows that the protocol of Liao is vulnerable to the impersonation attack. Peeters et al. have demonstrated in [119] that during Liao et al. protocol, an attacker can easily find $Z_{T}$, the secret identity of the tag. This can be done if the attacker sends the value $=-P_{S}$ to the tag. The latter will respond by sending the quantity $=-r_{1}{}_{}+P_{S}$, which is automatically equal to $Z_{T}$. As a result, Peeters et al. have proven that this protocol cannot resist the tracking attack. To justify this hypothesis, Zhao et al. in [109] have shown that if an attacker generates a random number $r_{2}$ and calculates $=P-P_{S}$, after receiving the quantities $Auth_{T}$ and $R_{1}$ calculated by the tag, he determines the value of $Z_{T}$ by computing $Auth_{T}-r_{2}R_{1}$, which gives:

$$\begin{array}{cc} Auth_{T}-r_{2}R_{1}= & Z_{T}+TK_{T1}+TK_{T2}-r_{2}\cdot R_{1}=Z_{T}+r_{1}R_{2}+r_{1}P_{S}-r_{2}R_{1}\\ & =Z_{T}+r_{1}\left(r_{2}P-P_{S}\right)+r_{1}P_{S}-r_{2}r_{1}P\\ & =+r_{2}P\;–\;r_{1}P_{S}+r_{1}P_{S}-r_{2}r_{1}P=Z_{T} \end{array}$$

In this way, Zhao shows that its protocol solves the key compromise problem that suffers Liao’s protocol. It means that if an attacker chooses an $r_{2}$ and calculates $=P-P_{S}$ then sends $R_{2}$ to the tag, then the tag will send in turn the values $R_{1}$ and $Auth_{T}$. This time the attacker cannot extract the value of the $Z_{T}$. This can be explained by the equation below. If the adversary wants to calculate $Auth_{T}-r_{2}R_{1}$ he will find:

$$\begin{array}{ll} Auth_{T}-r_{2}R_{1}= & Z_{T}+TK_{T1}+TK_{T2}-r_{2}R_{1}\\ & =Z_{T}+(r_{1}K_{x})R_{2}+(r_{1}K_{y})P_{s}-r_{2}R_{1}\\ & =Z_{T}+(r_{1}K_{x})r_{2}P-P_{s})+(r_{1}K_{y})P_{s}-r_{2}r_{1}P\\ & =Z_{T}+r_{1}K_{x}r_{2}P-r_{1}K_{x}P_{s}+r_{1}K_{y}P_{s}-r_{2}r_{1}P \end{array}$$

The attacker cannot apply the same scenario used to strike Liao’s protocol to extract the value of $Z_{T}$.

As we can see from Table 7, Alamr’s protocol is effective against MITM attacks. In fact, this protocol is totally secure against the three main attacks (MITMA, replay attack and impersonation attack). To prove this security, Alamr makes a reasonable assumption:

✓ All random numbers used are refreshed at each session.
✓ The private key of the tag is kept secret and known only by the tag itself.
✓ The private key of the reader is kept secret and known only by the reader itself.

It is also shown in Table 7 that the protocol of Dinarvand has a weakness against de-synchronization attacks. In fact, Dinarvand’s protocol has an updating phase for $ID_{S}$ and K values to prevent desynchronization attacks. To achieve this goal, Dinarvand has indicated that the server must keep the old and new $ID_{S}$ values for each session. However, updating of this value is done by the server itself and at the last step of the protocol. If, therefore, an attacker intervenes to block the latest message sent by the tag, the server is unable to update its $ID_{S}$ value. In this way, the protocol becomes vulnerable to the desynchronization attack. In addition, the attacker can easily extract the tag identifier $x_{T}$, since it is sent in the clear to the server. This allows the attacker to trace the user’s location using the tag identifier.

Benssalah has shown that Naeem’s protocol has some security weaknesses and vulnerabilities to some wireless attacks. In fact, he showed that the tag identity could be known by an attacker through the following process:

✓ If an attacker chooses a random number $r_{1}=1$, he can present himself as a legitimate reader and send $R_{1}=r_{1}P$ to the tag.
✓ When the tag receives the value of $R_{1}=P$, it calculates $T_{1}=t_{1}P$, $C_{1}=t_{1}R_{1}$ and $C_{2}=X_{T}+h\left(T_{1},R_{1},C_{1}\right)$ and sends the values $C_{1}$ and $C_{2}$ to the attacker.
✓ This way, the attacker calculates $X_{T}=C_{2}-h\left(T_{1},R_{1},C_{1}\right)=C_{2}-h\left(C_{1},P,C_{1}\right)$ since both quantities $C_{1}$ and $C_{2}$ have been publicly sent.

The attacker can therefore obtain the tag identity and present himself as a legitimate reader.

Once the identity of the tag has been successfully extracted, this protocol will be vulnerable to tag impersonation attacks. In fact, during a new authentication session, when the legitimate reader sends ${R_{1}}^{\prime }={r_{1}}^{\prime }P$ to the tag, the attacker reacts and intercepts this message. Then, the attacker generates a random number ${t_{1}}^{\prime }$ and calculates ${T_{1}}^{\prime }={t_{1}}^{\prime }P$, ${C_{1}}^{\prime }={t_{1}}^{\prime }{R_{1}}^{\prime }$, and ${C_{2}}^{\prime }=X_{T}+h\left({T_{1}}^{\prime },{R_{1}}^{\prime },{C_{1}}^{\prime }\right)$ and reply to the reader by sending ${C_{1}}^{\prime }$ and ${C_{1}}^{\prime }$. The reader, this time, calculates ${T_{1}}^{\prime }$ and determines the value $X_{T}$. Then, it compares the value of $X_{T}$ found to the one stored in its database and authenticates the attacker as the legitimate tag.

Zheng showed that if an attacker can get the values $R_{1}$, $R_{2}$, AT, and $AT^{\prime }$, he cannot be able to calculate $S_{T}R_{1}$ neither $S_{S}R_{2}$, since the values of $S_{T}$ and $S_{S}$ are kept secret, and they are known, respectively, only by the tag and the server. For example, the server determines the value of $P_{T}$ from its private key $S_{S}\left(P_{T}=AT-S_{S}.R_{2}\right)$. For an attacker, since it does not have the value of $S_{S}$, it cannot know the value of $P_{T}$ and, therefore, it cannot identify the tag. In the quantity $AT^{\prime }=S_{T}R_{1}-r_{2}R_{1}$, the value $S_{T}R_{1}$, which is assumed to be the tag signature information, is encrypted by $r_{2}.R_{1}$. To obtain this signature, the attacker needs to solve the discrete logarithm problem to calculate $r_{2}R_{1}$ from $R_{1}$.

For the protocol of Benssalah, it is mentioned in Table 7 that this protocol offers an efficient security to the different attacks: tracking attack, man-in-the-middle attack, de-synchronization attack, replay attack, impersonation attack.

From the protocol of Yang, we can see that the tag’s ID identity and its private key $x_{T}$ are included in the messages $M_{1}$ and $M_{2}$, where $Pid_{1}=H\left(ID,ts_{1}\right)$ and $Pid_{2}=H\left(X_{T},ID,ts_{2}\right)$. So, the identity of the tag is known only by the tag and the server. The attacker cannot determine the value of the secret key $x_{T}$ from the key $X_{T}$ because of the difficulty of the discrete logarithm problem. In addition, Yang has shown the effectiveness of the protocol against replay attacks since the protocol uses a time variable ts. Indeed, if there is a replay message, the value of ts will automatically exceed the expiration times set by the service.

According to Aloui’s protocol, the tag receives a random message $R_{r}$ from the reader without checking the validity of this request. The tag must wait to finish the whole authentication process in order to decide on the legitimacy of the authentication request. In this way, the tag cannot block the number of potential requests or control interrogations from unauthorized readers. As a result, the protocol may be vulnerable to denial of service attacks.

Arslan et al. analyzed in their paper [121] the security of the Izza protocol and showed that this protocol suffers from desynchronization attacks. Even if the scheme does not suffer from a denial of service attack, it does not provide authentication between the tag and the reader because of the use of the $PID_{T_{i\;old}}$ and $PID_{R_{\;old}}$ values. Indeed, the old $PID_{T_{i\;old}}$ and $PID_{R_{\;old}}$ values are not updated on the tag and reader side, respectively. In order to avoid these synchronization problems, Arslan has proposed some modifications that consist in updating the $PID_{T_{i\;old}}$ of the tag with the same mechanism as the one used on MS side.

Finally, we can conclude from what has been published until now that, Zheng [114], Zhao [109] and Benssalah’s [113] protocols offer excellent security against the various wireless attacks as presented in Table 7. This implies the security effectiveness of these three proposed protocols. 

#### 7.2.2. Security against Side-Channel Attacks

The security analysis of each protocol against SCA attacks were presented in Table 8. In this part of the paper, we will detail the security study of each protocol with respect to SCA attacks. 

All research works published in the literature, show that side-channel attacks, especially differential attacks, target the power consumption or the electromagnetic field variation between the tag and the reader to determine the secret keys shared during the communication. As mentioned in Section 3.5, the vulnerability of the RFID protocols previously described against side-channel attacks relies on the strength of the implemented cryptographic encryption primitives and the randomization of the processed data. As shown in Table 4, all the proposed protocols, except the Yang’s protocol, generate secret random numbers for the tag and the server ($r_{1}$ and $r_{2}$). Consequently, the quantities shared between the tag and the server will be modified at each round and the attacker cannot find the link between the collected power traces and the processed data. This way, the implementation of the DPA and DEMA attacks cannot determine the values of the random numbers used in the encryption of the data shared between the tag and the server during the communication. However, the secret keys that are stored in the tag and server databases always remain sensitive to side-channel attacks, as long as the encryption primitive used is not protected against these attacks. Table 8 summarizes the vulnerability of the different protocols presented to SCA attacks, in both transmitted data and the secrets stored in their databases.

For example, in the Liao protocol [24], the scalar multiplication algorithm used is the Montgomery ladder, which is assumed to be an effective countermeasure to the simple power analysis (SPA) attack [122]. Thus, as noted in Table 8, the Liao protocol is effective against SPA attacks, but is not proven to be secure against DPA and DEMA attacks since the Montgomery ladder algorithm always remains susceptible to DPA and DEMA attacks. As mentioned in Liao’s protocol authentication phase in Section 6.1.2, the calculation of the quantities $TK_{T1}$, $TK_{T2}$, $TK_{S1}$, and $TK_{S2}$ is performed based on random numbers $r_{1}$ and $r_{2}$. This way, the values of these quantities change at each new authentication session. Consequently, these quantities shared between the tag and the server cannot be targeted by DPA and DEMA attacks. On the other hand, the tag’s secret key $x_{T}$ can be the target of a DPA attack by knowing the scalar multiplication algorithm used and by performing several executions of the scalar multiplication operation with different values of P.

The Zhao protocol is an improvement of the Liao protocol, it uses the same scalar multiplication algorithm and has the same computational performance as the Liao protocol. For this reason, as shown in Table 8, both protocols have the same security weaknesses against side-channel attacks.

The authentication phase of the Alamr et al. protocol uses the elliptic curve Diffie-Hellman key exchange protocol (ECDH) to construct the secret keys shared between the tag and the server. In fact, Coron et al. showed in their paper [123] that implementations of elliptic curve protocols, such as El-Gamal encryption or Diffie-Hellman key exchange, are vulnerable to differential power analysis attacks if they are not properly protected. In the case of the Alamr’s protocol, the keys exchanged between the tag and the server, $Tk_{ag}$ and $Rk_{ag}$, are based on randomly generated numbers $t_{1}$ and $r_{2}$ in such a way that the values of these shared keys will be changed at each new authentication session. For this reason, we can state that the secret keys shared between the tag and the server during the authentication of the Alamr’s protocol are well protected against the DPA and DEMA attacks, while the security of this protocol against the SPA attack depends on the efficiency of the scalar multiplication algorithm used.

For Dinarvand’s protocol, the secret key K shared between the tag and the server and the pseudonym $ID_{S}$ change at each iteration during the updating phase. This feature prevents the effective implementation of DPA and DEMA attacks. Indeed, as mentioned in Section 3.2, changing the scalar at each execution reduces the chances of effective implementation of DPA attacks (same for DEMA). Even the authentication messages, $Auth_{S}$ and $Auth_{T}$, are computed based on random numbers $r_{1}$ and $r_{2}$, which reduces the risks of vulnerability of these messages to DPA and DEMA attacks.

For Yang, Naeem, and Benssalah’s protocols, the security of the transmitted data between the tag and the server against side-channel attacks depends on the effectiveness of the hash function used for encryption. However, as proven by Hoerder et al. in [124], since the operations performed during the execution of H function depend on a fixed security-critical input, such as ID, the computation process is vulnerable to SPA attacks. In addition, Hoerder showed in [124] that DPA (same for DEMA) attack is possible when the input of an H function combines security-critical fixed data with variable data that can be controlled by the attacker. That is, the input call looks like H(s, m) for a fixed security-critical s, and a variable m. This is the case in the Yang protocol [115] during the calculation of $Pid_{1}=H\left(ID,ts_{1}\right)$, where ID is the security-critical fixed quantity, and $ts_{1}$ is the variable known by the attacker. The same thing for the calculation of the quantity $Ver_{S}=H\left(ts_{1},Pid_{1},Aut{h_{S}}^{\prime }\right)$. For the protocol of Naeem, the quantity $C_{4}=h\left(C_{3},X_{T},T_{1},R_{1}\right)$ combines security-critical fixed data, $X_{T}$, with variable data that can be controlled by the attacker such as $R_{1}$. In addition, the hach function used to calculate $R_{5}=h\left(x_{T}\vee \left|{\left\{R_{2}\right\}}_{x}\right|\vee r_{1}\vee R_{4}\right)$ in the protocol of Benssalah combine the tag identity $x_{T}$ and the random number $r_{1}$, wich can be fixed by the attacker. We can conclude that the protocols of Yang, Naeem, and Benssalah present SCA vulnerability during the data transmission.

Aloui uses, in the two scalar multiplication operations performed by the tag, the random number $r_{n}$. In this way, the calculation of these operations is supposed to be secure against side-channel attacks. For the hash operations, the computed quantity $H(\left(r_{n},K_{n2}\right)\left(Q_{r},R_{r}\right)=\left(h_{r1},h_{r2}\right)$ as well as the value of $H(id_{n}\left|\left|K_{n1}\right|\right|K_{n2}\left|\left|R_{r}\right|\right|R_{n}||h_{r2})=(h_{1}||h_{2})$ depend immediately on the value $r_{n}$, and do not satisfy the conditions for success of DPA described by Hoerder [124] on such functions. On the other hand, the scalar multiplication operation $Q_{r}=d_{r}.G$ performed by the reader can be targeted by SPA and DPA attacks if the encryption primitive used is vulnerable against these types of attacks. Although the data transmitted between the tag and the reader are protected against SCA attacks, the secret keys stored in the reader’s database remain vulnerable to these types of attacks.

During the Step 2 of Izza’s protocol, the inputs of the first hash function, computed in $PID_{T_{i\;new}}=h(PID_{T_{i\;old}}||init),$ combine the pseudo tag identity $ID_{T}$ and the random variable init. The second value $C_{2}=PID_{T_{i\;new}}+h({\left(R_{t1}\right)}_{x}\left|\left|{\left(R_{r1}\right)}_{x}\right|\right|{\left(C_{1}\right)}_{x}||T_{1})$ performs the hash operation between the values $(R_{t1}$ and $C_{1})$ generated by the tag and the public value $R_{r1}$. Consequently, since the init and $R_{r1}$ values can be manipulated by an attacker, these two hash operations can be the target of a SCA attack in order to extract the pseudo identity of the tag.

We can therefore deduce from the results found in Table 8 that the security of RFID protocols against SPA attacks depends essentially on the efficiency of the scalar multiplication algorithm used. For DPA attacks, there are two types of vulnerability of these proposed protocols; the vulnerability of the data transmitted between the tag and the server that can provide the attacker to listen to and modify the communication, and the vulnerability of the private keys that are already stored in the databases of the entities communicating together. To protect an ECC-based RFID authentication protocol well, it is first necessary to carefully choose the scalar multiplication algorithm used to avoid any consumption leakage and to unify the numbers of the operations used. Secondly, it is required to randomize the secret data used during the communication as well as those stored in the entities’ database.

#### 7.2.3. Security Requirements 

Table 9 examines the effectiveness of the proposed protocols in providing security requirements. As we can see from this table, in addition to mutual authentication, the protocol of Alamr ensures confidentiality, anonymity, forward security, and location privacy. However, it cannot ensure scalability or availability which is not desirable in IoT environments. This claim is explained in the paper of Naeem et al. [111] where the author indicated that the reading scheme proposed by Alamr is dedicated to a single tag, whereas in general cases an RFID reader is supposed to work with hundreds or even thousands of tags.

It can be noted that Zhao’s protocol cannot ensure all security services. Indeed, it cannot assure data integrity [125]. It means that the reader cannot detect if there is any modification or falsification of the data received by the tag. The protocol of Dinarvand, which is the least expensive protocol in terms of the number of operations, cannot ensure the location privacy service. As indicated in the protocol’s authentication phase (Section 4.4.2), the tag identification is clearly transmitted to the server. Thus, an attacker can easily find the location of the tag using the tag identity. 

Regarding security features, Arslan has shown in his paper [121] that the protocol proposed by Izza et al. suffers particularly from the existing relationship between the $C_{3}$ message and the long-term identity of the tag $ID_{T}$. Therefore, this protocol cannot offer privacy, including tag anonymity or forward secrecy.

All the proposed protocols are based on elliptic curves, and all ensure mutual authentication between the tag and the reader. From all the comparative tables we can conclude that the protocol of Zheng and the protocol of Benssalah present the most secure authentication protocol in term of security features. On the other hand, as stated in Section 7.1, the Benssalah’s protocol requires more expensive operations.

The Dinarvand’s protocol does not provide perfect security against the attacks listed in Table 7, but it is very effective in terms of cost and based on Table 8 it offers the best security against side-channel attacks. To select the best ECC-based RFID authentication protocol, it is necessary to take into account the security of the data transmitted and the resource limitations of the RFID tags. For this reason, the protocol of Zheng presents a good compromise between the number of operations required, the computation cost, the communication cost, and the security against the various proposed attacks. 

## 8. Conclusions and Perspectives

The objective of this paper was to perform a comparative study between ECC-based RFID authentication protocols in terms of security and performance. Our survey presented the authentication protocols published between 2014 and 2021. To achieve our goal, we started first by citing and explaining the different attacks that could suffer an RFID protocol. We divided these attacks into wireless attacks that aimed to intercept the tag-server communication and hardware attacks that targeted the cryptographic primitives used in the protocol. Secondly, we presented the different lightweight ECC implementations dedicated to RFID tags. We mentioned the various methods used in the literature to minimize the area required for scalar multiplication calculation. These methods allowed to design ECC hardware architectures that met the limited resource constraints of RFID tags. Then, we made a detailed explanation of each published protocol, giving the advantages and disadvantages of each one. Finally, by reviewing the different published results, a comparative study was carried out between these different works in terms of performance and security. Since all these proposed protocols used ECC as algorithms, and since SCA attacks were one of the most popular hardware attacks against such cryptosystems, we studied the vulnerability of each proposed protocol to these kinds of attacks. There were other types of hardware attacks, called fault attacks (FA), which were effective against ECC-based cryptosystems. In future work, we will study the vulnerability of the ECC implementations used in RFID authentication protocols to FA. Finally, we will aim to implement a countermeasure method for SCA and FA attacks to ensure perfect security for an RFID protocol, taking into account the limited resources and the limited consumption of RFID tags. 

## Figures and Tables

**Table 1:** Classification of the operating frequencies of RFID tags [34].

Nomination	Frequency	Read Ranges	Type of Tag	Cost	Application
*LF*	125–134 Khz	10–150 cm	Passive	Low	Animal identification
*HF*	13.56 Mhz	Up to 5 m	Passive	Low	Access control
*UHF*	433–960 Mhz	Up to 10 m	Passive\active	High	Logistics, stock management

**Table 2:** Coordinate systems used by research works.

Coordinates	[88]	[89]	[90]	[91]	[92]	[93]	[94]	[95]	[96]	[97]	[98]	[99]
Affine coordinates			√	√							√	
Standard projective							√					
Jacobian projective		√										
Lopez and Dahab	√				√	√		√	√	√		√

**Table 3:** Implementation results of described works.

Work		Curve		Tech [µm]	Area [Gate]	Power [µW]	Cycles	Energy [µJ]
Batina [25]		B-131	d = 1	0.18	6718	Under 30	210,600	-
			d = 2		7191		109,200	
			d = 3		7645		74,880	
			d = 4		8104		57,720	
		B-163	d = 1	0.18	8214	-	353,710	-
			d = 2		8791		182,071	
			d = 3		9368		124,858	
			d = 4		9926		95,159	
Lee [103]		B-163	d = 1	0.13	12,506	36.63	275,816	8.94
			d = 2		14,064	21.55	144,842	5.29
			d = 3		14,729	15.75	101,183	3.88
			d = 4		15,356	12.08	78,544	2.94
Wenger [26]		B-163		UMC L130	8958	32.34	286,000	9.25
Wenger [27]	Ar1	B-163		0.13	14,167	49.1	7,216,905	354.3
	Ar2				11,778	93.8	342,724	32.1
	Ar3				4114	66.1	467,370	30.9
Roy [106]		K-283		0.13	4323	6.11	1,566,000	9.56

**Table 4:** Operations requirement of each proposed protocol.

Requirement	Liao		Zhao		Alamr		Naeem		Dinarvand		Benssalah		Zheng		Yang		Aloui		Izza	
	T	R	T	R	T	R	T	R	T	R	T	R	T	R	T	R	T	R	T	R
Random number	1	1	1	1	1	2	1	2	1	1	1	1	1	1	0	0	1	1	1	1
Scalar multiplication	5	5	5	5	4	5	5	5	3	3	3	1	4	4	1	1	2	2	2	4
Point addition	2	2	2	2	1	1	1	1	0	0	1	1	3	3	0	0	0	0	0	0
XOR operation	0	0	0	0	0	0	0	0	2	2	0	0	0	0	0	0	2	2	1	1
Hash function	0	0	0	0	0	0	2	2	0	0	3	3	0	0	4	4	2	1	6	7

T: tag, R: reader.

**Table 5:** Computation time comparison.

Protocol		Computation Time (ms)	
	Tag	Reader	Total
Liao [24]	64 × 5 = 320	64 × 5 = 320	640
Zhao [109]	64 × 5 = 320	64 × 5 = 320	640
Alamr [110]	64 × 4 = 256	64 × 5 = 320	576
Naeem [111]	64 × 5 + 2 × T_H_ ^1^ = 320 + 2 × T_H_ ^1^	64 × 5 + 2 × T_H_ ^1^ = 320 + 2 × T_H_ ^1^	640 + 4 × T_H_ ^1^
Dinarvand [112]	64 × 3 = 192	64 × 3 = 192	384
Benssalah [113]	64 × 3 + 3 × T_H_ ^1^ = 192 + 3 × T_H_ ^1^	64 + 3 × T_H_ ^1^	256 + 6 × T_H_ ^1^
Zheng [114]	64 × 4 = 256	64 × 4 = 256	448
Yang [115]	64 + 4 × T_H_ ^1^	64 + 4 × T_H_ ^1^	128 + 8 × T_H_ ^1^
Aloui [116]	689.32	75.88	765.20
Izza [117]	64 × 2 + 6 × T_H_ ^1^ = 128 + 6 × T_H_ ^1^	64 × 4 + 7 × T_H_ ^1^ = 256 + 7 × T_H_ ^1^	384 + 13 × T_H_ ^1^

^1^ The time of executing one Hash operation.

**Table 6:** Communication cost comparison.

Protocol	Communication Cost (bits)		
	Tag	Reader	Total
Liao [24]	640	640	1280
Zhao [109]	640	640	1280
Alamr [110]	640	960	1600
Naeem [111]	480	480	960
Dinarvand [112]	800	640	1440
Benssalah [113]	320	480	800
Zheng [114]	640	640	1280
Yang [115]	224	224	448
Aloui [116]	768	512	1280
Izza [117]	1280	1280	2560

**Table 7:** Resistance to wireless attacks [120].

Attacks	Liao [24]	Zhao [109]	Alamr [110]	Naeem [111]	Dinarvand [112]	Benssalah [113]	Zheng [114]	Yang [115]	Aloui [116]	Izza [117]
MITMA	Yes	Yes	Yes	Yes	Yes	Yes	Yes	-	-	-
Replay	Yes	Yes	Yes	Yes	Yes	Yes	Yes	Yes	Yes	-
Impersonation	No	Yes	Yes	No	No	Yes	Yes	Yes	Yes	-
Key compromise	No	Yes	Yes	Yes	No	Yes	Yes	-	-	-
Location tracking	Yes	Yes	Yes	Yes	Yes	Yes	Yes	-	-	-
DoS	Yes	Yes	No	Yes	Yes	Yes	Yes	Yes	No	Yes
Cloning	Yes	Yes	Yes	Yes	Yes	Yes	Yes	Yes	-	-
Server spoofing	Yes	Yes	Yes	Yes	Yes	Yes	Yes	-	-	-
De-synchronization	Yes	Yes	No	Yes	No	Yes	Yes	-	Yes	No

Yes: secure against such attacks. No: not secure against such attacks. -: untreated.

**Table 8:** Resistance to side-channel attacks.

Attacks		Liao	Zhao	Alamr	Naeem	Dinarvand	Benssalah	Zheng	Yang	Aloui	Izza
**Security of Transmitted Data**											
*SCA*	*SPA*	-	-	-	No	-	No	-	No	Yes	No
	*DPA*	Yes	Yes	Yes	No	Yes	No	Yes	No	Yes	No
	*DEMA*	Yes	Yes	Yes	No	Yes	No	Yes	No	Yes	No
**Security of Secret Keys**											
*SCA*	*SPA*	Yes	Yes	-	-	-	-	-	-	-	-
	*DPA*	No	No	No	No	Yes	No	No	No	No	No
	*DEMA*	No	No	No	No	Yes	No	No	No	No	No

**Table 9:** Security features analysis.

Security Service	Liao [24]	Zhao [109]	Alamr [110]	Naeem [111]	Dinarvand [112]	Benssalah [113]	Zheng [114]	Yang [115]	Aloui [116]	Izza [117]
Confidentiality	Yes	Yes	Yes	Yes	Yes	Yes	Yes	Yes	-	-
Availability	Yes	Yes	No	Yes	Yes	Yes	Yes	-	Yes	-
Forward secrecy	Yes	Yes	Yes	-	Yes	Yes	Yes	-	Yes	No
Mutual authentication	Yes	Yes	Yes	Yes	Yes	Yes	Yes	Yes	Yes	Yes
Anonymity	Yes	Yes	Yes	Yes	Yes	Yes	Yes	Yes	Yes	No
Scalability	Yes	Yes	No	Yes	Yes	Yes	Yes	-	Yes	Yes
Location privacy	Yes	Yes	Yes	Yes	No	Yes	Yes	-	-	No
Data integrity	Yes	No	No	Yes	Yes	Yes	Yes	Yes	-	-

## Data Availability

Not applicable.
